# Interfacial cfDNA Enrichment and Amplification with On‐Chip Thermoplasmonics for Highly Sensitive Cancerous Liquid Biopsy

**DOI:** 10.1002/advs.202409708

**Published:** 2024-12-04

**Authors:** Danhua Wang, Linlin Liu, Wenjing Chi, Zhenping Liu, Jiayun Wu, Yirou Liang, Fei He, Ruixiang Zhang, Pengxin Huang, Yunbo Li, Guangyu Qiu

**Affiliations:** ^1^ Institute of Medical Robotics, School of Biomedical Engineering Shanghai Jiao Tong University Shanghai 200240 China; ^2^ Department of Laboratory Medicine Huadong Hospital Affiliated to Fudan University Shanghai 200031 China; ^3^ The First People's Hospital of Linping District Hangzhou Zhejiang Province 311100 China

**Keywords:** biosensors, cell‐free DNA, liquid biopsy, lung cancer, nucleic acid amplification, thermoplasmonics

## Abstract

Tumor‐derived cell‐free DNA (cfDNA) has been exploited as an effective liquid biopsy biomarker for early cancer diagnosis. However, the fragmented and low‐abundance nature in circulating blood pose challenges for highly sensitive cfDNA quantification. Herein, a multifunctional plasmonic biosensor termed Interfacial cfDNA Enrichment, Amplification and Sensing with on‐chip Thermoplasmonics (INEAST) is developed for cfDNA‐based liquid biopsy and lung cancer diagnosis. The INEAST biosensor achieved in situ thermoregulation and label‐free cfDNA biosensing by simultaneously harnessing interfacial thermoplasmonics and localized surface plasmon resonance. Typical cfDNA biomarkers, including epidermal growth factor receptor (*EGFR)*, tumor protein 53 (*TP53)*, phosphatase and tensin homologue deleted on chromosome 10 (*PTEN)*, and cyclin‐dependent kinase inhibitor (*CDKN2A)*, are quantified with detection limits down to femtomolar‐level. Through further validation using blood samples from lung cancer patients, the proposed INEAST bioassays demonstrated superior reliability for lung cancer screening, particularly when combined with clinically available tumor‐protein metrics. This study demonstrated that the INEAST biosensor enables rapid and sensitive cfDNA quantification, yielding a promising and compatible liquid biopsy for early‐stage lung cancer diagnosis.

## Graphical abstract

This study established a multifunctional plasmonic biosensor entitled Interfacial cell‐free DNA (cfDNA) Enrichment, Amplification, and Sensing using on‐chip Thermoplasmonics (INEAST) for cfDNA‐based liquid biopsy and lung cancer diagnosis. By harnessing interfacial thermoplasmonics and localized surface plasmon resonance, the INEAST system can in situ monitor cfDNA hybridization and amplification in a real‐time and label‐free manner. This work provides novel insights into on‐chip thermoplasmonic biosensors for cfDNA quantification and offers a promising liquid biopsy for early‐stage lung cancer diagnosis.

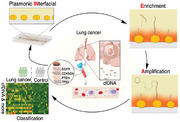

## 1 Introduction

Early diagnosis of malignant tumors is critical for improving clinical consequences and patient survival. Currently, the overall 5‐year survival rate of lung cancer is less than 20%.^[^ ^1^ ^]^ Among them, up to 85% of these patients were diagnosed at an advanced or metastatic stage, with a 5‐year survival rate of only ≈2%.^[^ ^2^ ^]^ In contrast, the postoperative 5‐year survival rate with carcinoma diagnosed at an early stage is approaching 100%.^[^ ^3^ ^]^ Liquid biopsy based on tumor biomarker detection in the bloodstream has been proven to be an effective strategy for early diagnosis. Among them, circulating cell‐free DNA (cfDNA) in humoral circulatory systems like the bloodstream has been considered a valuable noninvasive liquid biopsy biomarker for cancer diagnosis and prognosis.^[^ ^4^ ^]^ Due to inefficient infiltration, cfDNA levels in cancer patients are significantly elevated compared to those of healthy individuals.^[^ ^5^ ^]^ Therefore, quantification of alterations in circulating cfDNA levels allows for precise and noninvasive cancer screening, diagnosis, and prognosis. However, recent studies have indicated that the circulating cfDNA has a short half‐life ranging from 35 to 230 min, with an average of 120 min.^[^ ^6^ ^]^ Therefore, it is imperative to develop diagnostic tools with high sensitivity and rapid detection capabilities to enhance diagnostic precision and early cancer detection.

Nucleic acid amplification testing (NAAT) currently plays a critical role in cfDNA‐based early cancer diagnostics due to its excellent sensitivity and simplicity. Although polymerase chain reaction (PCR) has been considered the gold‐standard NAAT approach, it has several drawbacks when it comes to detecting small fragments of nucleic acid, including cfDNA.^[^ ^7^ ^]^ For example, PCR‐based amplification and quantification of these short cfDNA fragments with 160–180 base pairs in length presents challenges related to primer design, potentially resulting in inaccuracies in quantitative analysis.^[^ ^8^ ^]^ Temperature‐dependent PCR amplification is also technically demanding for high‐precision temperature control instruments,^[^ ^9^ ^]^ thus limiting its application in resource‐limited areas. Isothermal NAAT approaches, such as rolling circle amplification (RCA), have shown promise for rapid, cost‐effective, and easy‐to‐use diagnosis.^[^ ^10^ ^]^ Although isothermal RCA methods offer advantages in terms of simplicity and specificity, conventional RCA‐based detection tools require separate pre‐amplification processes and lack the ability to perform swift, real‐time, and high‐precision quantitative assays, thus limiting their practical applications. In addition, accurately identifying the amplification endpoint for conventional NAAT bioassays also poses difficulties for highly precise cfDNA quantification.

Interfacial localized surface plasmon resonance (LSPR) is a label‐free biosensing technology that harnesses light‐matter interactions to harvest photon energy and construct local electromagnetic fields for highly sensitive biomolecular detection.^[^ ^11^ ^]^ Recent studies have revealed that plasmonic nanoparticles can be utilized as multifunctional photonic (MFP) media that simultaneously act as nanoabsorber, nanoheater, and nanotransducer for simultaneous nucleic acid thermoregulation and biosensing.^[^ ^12^ ^]^ Specifically, plasmonic nanotransducers within the LSPR biosensors can monitor localized nucleic acid hybridization and amplification reactions to provide sensitive responses for cfDNA quantification in a real‐time and label‐free manner. Meanwhile, the converted plasmonic photothermal (PPT) heat energy, also known as the thermoplasmonic effect that is highly localized in the vicinity of nanoparticles, can be harnessed as an in situ and highly precise heat source for near‐field thermal manipulation.^[^ ^12a,c^ ^]^ Therefore, thermoplasmonic heating has the potential to regulate local biological reactions, including DNA hybridization and amplification. In addition, the LSPR‐enhanced photothermal system may simultaneously excite the production of high‐energy charge carriers and heat energy, which potentially improves the kinetics of biochemical reactions for nucleic acid amplification and detection.^[^ ^13^ ^]^ Thus, MFP plasmonic biosensors that integrate the abilities of label‐free transduction and efficient thermoregulation of NAAT processes may serve as a promising liquid biopsy approach for highly sensitive cfDNA detection and early cancer diagnosis.

**Figure 1:**
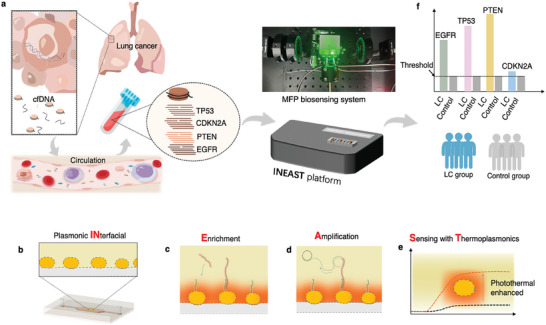
Schematic illustration of cfDNA‐based liquid biopsy with the INEAST bioassay. a) After apoptosis or necrosis of cancer cells, nuclease cleaves the long‐stranded DNA in the nucleus, resulting in the production of cfDNA. The tumor‐associated cfDNAs are present in significantly elevated levels in the bloodstream, which can be quantified by the proposed INEAST bioassays for liquid biopsy. b) Schematic illustration of AuNI biosensing chip and microfluidics for plasmonic interfacial amplification and sensing within the INEAST system. c) Selective cfDNA enrichment on INEAST for highly sensitive detection. d) Schematic illustration of the interfacial and on‐chip cfDNA amplification with INEAST‐based thermoplasmonics. e) The thermoplasmonic effect within INEAST system enhanced the cfDNA biosensing responses. f) Multiple tumor‐associated indicators, retrieved by the proposed INEAST‐based cfDNA bioassays, were employed for LC diagnosis and screening applications.

In this study, we developed a photonic biosensing platform termed Interfacial cfDNA Enrichment, Amplification, and Sensing with on‐chip Thermoplasmonics (INEAST) for cfDNA‐based liquid biopsy and lung cancer diagnosis (**Figure** **1**). This biosensing platform demonstrated a thermoplasmonic‐mediated NAAT approach for analyzing multiple cfDNA biomarkers in clinical plasma samples. The initial cfDNA hybridization and subsequent isothermal amplification reaction on the solid‐phase LSPR sensor chips can be real‐time monitored by the proposed INEAST system in a label‐free and highly sensitive manner. Moreover, we demonstrated that the optimized interfacial thermoplasmonic effect significantly enhanced the cfDNA detection sensitivity without requiring additional readout labeling. Through further clinical validation with patient blood samples, we demonstrated that the INEAST liquid biopsy method can provide tumor load information for lung cancer classification and diagnosis. Notably, the INEAST platform exhibited substantial utility for reliable LC diagnosis when combined with clinically available glycomarker bioassays.

## 2 Results

### 2.1 Working Principle of INEAST for Cancerous cfDNA Detection

The INEAST workflow consisted of on‐chip regulation of targeted cfDNA via the MFP‐LSPR matrix, in which interfacial cfDNA enrichment, local thermoplasmonic amplification, and label‐free LSPR biomarker transduction were sequentially achieved in succession as demonstrated in **Figure** **1**. The INEAST platform took advantage of two major functional plasmonic effects, as depicted in Figure 1b–d and Figure S1 (Supporting Information). The plasmonic photothermal stimulation unit, as shown in Figure S1a (Supporting Information), allowed for precise local temperature control and regulation of nucleic acid amplification at the biosensing interface. The homogenized laser with 532 nm peak wavelength was applied to generate a stable and intense thermoplasmonic field. Additionally, the differential phase‐sensitive LSPR transduction unit, as shown in Figure S1b (Supporting Information), allowed for real‐time and label‐free cfDNA quantification. An attenuated total reflection‐based broadband light source was applied to excite collective free electron oscillations for label‐free monitoring of the interfacial cfDNA sequence hybridization, enrichment (Figure 1c), and amplification (Figure 1d). The 2D gold nanoislands (AuNIs) on the biosensing interface, as shown in Figure S1c–e (Supporting Information) and Figure 1b–d, were employed as MFP media for on‐chip thermoplasmonics and LSPR biosensing. The peak absorption wavelength of AuNI was optimized to 532.2 nm (± 0.2 nm), which perfectly aligns with the laser excitation to achieve high photothermal conversion efficiency and precise interfacial temperature manipulation (Figure S2, Supporting Information). By harnessing the fine‐tuned thermoplasmonics, highly efficient cfDNA amplification can be achieved, thus significantly enhancing the biosensing responses and achieving outperformed sensitivity as shown in Figure 1e.

For practical cfDNA biosensing applications, the on‐chip INEAST bioassay required four essential steps, including biosensor functionalization, surface blocking, direct cfDNA hybridization, and interfacial on situ amplification, as shown in Figure S3 (Supporting Information). These processes induced alterations of the local refractive index, resulting in detectable plasmonic phase changes that can be detected in real‐time as shown in Figure S3a–d (Supporting Information). Using the INEAST platform, the targeted cfDNA sequences that were extracted in blood samples were successively quantified through both the on‐chip hybridization‐based and amplification‐based INEAST bioassays for accurate quantitative analysis as shown in Figure 1f. We aimed to enhance the understanding of the correlation between the cfDNA expression levels and their relationship across the diagnosis model (Figure 1f). The related DNA sequences, including the complementary DNA (cDNA) capture sequences for hybridization‐based cfDNA enrichment and the circular DNAs for thermoplasmonic‐based cfDNA amplification, were summarized in Table S1 (Supporting Information).

### 2.2 Optimization and Characterizations of the INEAST Platform

**Figure 2:**
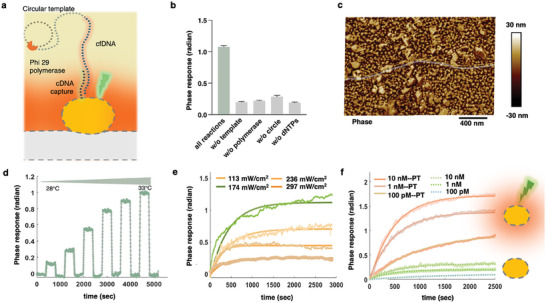
Validation and performance optimization of the cfDNA‐triggered INEAST bioassays. a) Schematic illustration of cfDNA‐triggered in situ hybridization and amplification on plasmonic AuNI. b) Biosensing validations of the INEAST amplification processes with reactant‐deficient groups. c) AFM phase mapping of the INEAST amplified DNA strands on AuNI biosensors interface. d) Real‐time characterization of the thermoplasmonic‐induced LSPR phase responses by switching the irradiated laser powers. e) The INEAST‐based cfDNA amplifications were real‐time characterized and optimized by testing 100 pm *EGFR*‐cfDNA under different thermoplasmonic heating conditions. f) Comparison of the biosensing enhancement when detecting different cfDNA concentrations by employing the optimal INEAST interfacial thermoplasmonic condition.

The amplification‐based INEAST bioassay was first characterized in terms of feasibility and specificity. The on‐chip INEAST amplification required four components, including the DNA polymerase, target cfDNA sequence, circular‐DNA template, and deoxynucleotide triphosphates (dNTPs).^[^ ^14^ ^]^ In the presence of the target cfDNA, the circular‐DNA template hybridized and subsequently initiated the cfDNA elongation to produce hundreds of tandem repeats by phi29 DNA polymerase (**Figure** **2**a). The amplification processes were validated with both agarose gel electrophoresis and INEAST real‐time biosensing approaches as shown in Figure 2b and Figure S4d (Supporting Information). Specifically, the presence of targeted cfDNA, i.e., the *EGFR* sequence, effectively initiated the RCA process in the bulk liquid phase and generated high molecular weight amplicons, which did not migrate out of the electrophoresis wells as shown in Figure S4d (Supporting Information). In contrast, the reactant‐deficient groups, which have the cfDNA, circular DNA templates, and dNTPs respectively absence in the reaction system, demonstrated no detectable process, Figure S4d (Supporting Information). The proposed INEAST platform was further validated in terms of specificity with on‐chip cfDNA amplification. When lacking necessary reactants, the INEAST biosensors reported weak LSPR phase responses at ≈0.24 radian as shown in Figure 2b. In contrast, with the presence of all reactants, INEAST effectively quantified the cfDNA amplification process and obtained a positive phase response at 1.1 radian.

The on‐chip cfDNA amplification with the INEAST method was further investigated with atomic force microscopy (AFM), as shown in Figure 2c and Figure S4b–d (Supporting Information). The surface roughness of the biosensor chips after INEAST amplification was quantified to be 2.94 nm, which was remarkably smooth compared to that of the bare AuNI biosensor chips at 3.26 nm (Figure S4a–c, Supporting Information). This indicated that the AuNI gaps on the INEAST sensing interface were filled with the immobilized capture sequences and the DNA amplicons. Furthermore, the interfacial INEAST amplicons, i.e., long interwound DNA strands, were also imaged as shown in the AFM scanning results (Figure 2c; Figure S4c, Supporting Information). The amplified cfDNA strands highlighted in the AFM images (Figure 2c) were found to be longer than 3 µm in length, indicating that the cfDNA length has been efficiently amplified to over 9k bp within the INEAST bioassay. These results directly demonstrated the feasibility of the proposed cfDNA amplification‐based INEAST bioassays.

In principle, the kinetics of nucleic acid binding and circular amplification are highly temperature‐dependent. Therefore, we further optimized the INEAST interfacial thermoplasmonic condition to enhance the cfDNA amplification efficiency and biosensor or sensitivity. Specifically, the local thermoplasmonic temperature and amplification efficiency were initial characterized to determine the optimal INEAST thermoplasmonic conditions. As the optical power density of the incident laser increased from 52.4 mW cm^−2^ to 237.6 mW cm^−2^, the LSPR phase responses of the thermoplasmonic biosensors increased from 0.12 to 1.00 radian, as shown in Figure 2d, which represented the designated elevation of interfacial temperature from 25.1 to 33.4 °C in the vicinity of the AuNI biosensing interface. The optimum PPT heating condition for the INEAST‐based cfDNA biosensing was subsequently analyzed by comparing the amplification efficiency under different thermoplasmonic conditions. As shown in Figure 2e, the 32.9 ^○^C thermoplasmonic heating environment constructed by the 174 mW cm^−2^ laser power density offered the best INEAST amplification performance, producing 1.1 radian LSPR phase responses when detecting 100 pm targeted cfDNA. In contrast, the amplification responses were ≈62.5% under 28.0 ^°^C with 91.2 mW cm^−2^ stimulation and ≈40.0% under 33.4 ^°^C with 237.6 mW cm^−2^, respectively.

The photothermal enhancements for INEAST amplification and quantification were further verified by testing cfDNA with varying concentrations as shown in Figure 2f. Accordingly, the INEAST responses were elevated to 0.8, 1.2, and 1.6 radian when employing optimal thermoplasmonic heating to quantify 100 pm, 1 nm, and 10 nm cfDNA specimens. In contrast, disabling the thermoplasmonic heating suppressed the amplification efficiencies, resulting in INEAST biosensing responses all below 0.3 radian. These results demonstrated significant enhancements for cfDNA detection by harnessing the interfacial thermoplasmonic effect. The cfDNA amplification efficiency with INEAST photothermal enhancement was 5.18 times higher for detecting 10 nm *EGFR* targets and 9.25 times for 100 pm *EGFR* compared to no thermoplasmonic conditions (Figure 2f; Table S2, Supporting Information). Therefore, we believe that the manipulated interfacial temperature and the boosted cfDNA amplification kinetics within the INEAST system are beneficial for the detection of trace amounts of target cfDNA sequences. Additionally, the localized heating potentially increased the rigidity of the cfDNA sequences, diminishing site‐blocking during the whole INEAST biosensing process.

### 2.3 Quantitative cfDNA Detection with INEAST Platform

Considering genetic heterogeneity of cancerous tumors, multiplexing detection of diverse cfDNA targets in liquid biopsy may mitigate test variability and enhance the reliability of cancer diagnosis. Therefore, four cfDNA targets were selected for INEAST‐based liquid biopsy due to their different LC regulatory pathways (Table S3, Supporting Information). Specifically, the LC commonly harbors alterations in the following three signaling pathways: 1) the receptor tyrosine kinase pathway, i.e., *RTK/RAS/PI3K*, 2) the tumor protein 53 (TP*53)* pathway, and 3) the retinoblastoma (*Rb)* pathway.^[^ ^15^ ^]^ The most frequently altered genes in the RTK/RAS/PI3K pathway include phosphatase *and tensin homologue deleted on chromosome 10 (PTEN)*, neurofibromatosis type 1 (*NF1)*, and *EGFR*.^[^ ^16^ ^]^ cyclin‐dependent kinase inhibitor 2A/B (*CDKN2A/B)* frequently undergoes alteration in the *Rb* pathway,^[^ ^17^ ^]^ while changes in the *TP53* gene potentially affect the *TP53* pathway.^[^ ^18^ ^]^ Therefore, cfDNA sequences including *PTEN*, *EGFR*, *CDKN2A/B*, and *TP53* from different LC regulatory pathways were considered as INEAST bioassay targets in this study.

**Figure 3:**
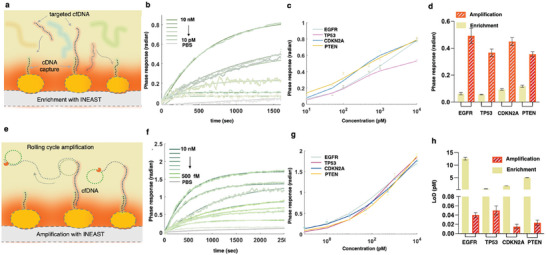
Characterizations of quantitative biosensing performance of cfDNAs interfacial enrichment and amplification in the INEAST system. a) Schematic diagram of the interfacial enrichment of targeted cfDNA with the INEAST. b) Real‐time sensorgrams of the direct *EGFR* cfDNA hybridization processes within the INEAST biosensors. c) Quantitative regression curves for the four targeted cfDNA sequences based on the enrichment bioassay. d) The comparison of INEAST phase responses induced by interfacial enrichment and amplification of the four different cfDNA targets at 10 pm concentrations. e) Schematic diagram of amplification‐based cfDNA detection with INEAST. f) Real‐time sensorgrams of the rolling cycle‐based amplification processes with INEAST bioassays for detecting 500 fM–10 nm *EGFR* cfDNA targets. g) Quantitative regression curves for the four targeted cfDNA sequences based on the INEAST amplification bioassay. h) The LoD comparison of the four cfDNA enrichment and amplification bioassays.

To assess the INEAST biosensing performance, the in situ cfDNA hybridization was further characterized through the phase‐sensitive LSPR system. As shown in **Figure** **3**a, the plasmonic AuNI transduced specific binding between the anchored cDNA capture sequence and the cfDNA targets, while the photothermal effect was optimized to enhance the binding kinetics and accuracy. The LSPR phase sensing responses toward various concentrations of the *EGFR*‐cfDNA sequences were real‐time monitored, as shown in Figure 3b. The surface enrichments of the other four cfDNA sequences, including *TP53*, *PTEN*, and *CDKN2A* cfDNAs, were real‐time quantified as shown in Figure S5a–h (Supporting Information). Based on these INEAST hybridization bioassay results, the Hill equation was employed as a regression fitting model to describe the surface enrichment process. Accordingly, the limit of detection (LoD) was calculated based on the International Union of Pure and Applied Chemistry definition as the sum of the blank measures, i.e., 2.92 × 10^−3^ radian with the PBS buffer and triple of their standard deviation. The INEAST plasmonic phase responses for detecting the four targeted cfDNAs at 10 pm were demonstrated in Figure 3d. Additionally, the limit of quantification (LoQ), which was considered tenfold of the standard deviation, was also calculated for evaluating their capability for quantitative cfDNA analysis. In detail, the hybridization‐based cfDNA detection within the INEAST system achieved a LoD down to 12.87 pm (7748 copies per µL) and a LoQ of 45.86 pm (27 608 copies per µL) for the *EGFR* cfDNA targets. Among them, the *TP53* cfDNA bioassay demonstrated the best LoD performance by 0.53 pm (319 copies per µL) (Figure S5g,h, Supporting Information). The Hill coefficiencies of these four cfDNA hybridization assays were found to be less than 1, which indicated negative cooperativities with the DNA binding process. Meanwhile, the results showed that the maximum *EGFR* response can reach 0.95 radian with a half‐maximum concentration at 761.2 pm (458 242 copies per µL) (Figure S5a,b, Supporting Information). The maximum *TP53* detection response was found to be only 0.69 radian, with a half‐maximum concentration of 252.8 pm (152 185 copies per µL) (Figure S5c,d, Supporting Information). The variations in the detection limits and regression features for the four cfDNA hybridization‐based assays may arise from differences in the sequence design and hybridization kinetics. Additionally, although the direct hybridization assays may not be particularly sensitive, they can be utilized as an effective cfDNA interfacial enrichment approach to facilitate subsequent local amplification for highly sensitive and label‐free INEAST quantification.

The amplification‐based INEAST bioassays were further investigated (Figure 3e) by quantifying the cfDNAs across a concentration range of 0.1 pm to 10 nm as shown in Figure 3f and Figure S5i–p (Supporting Information). Compared to the hybridization‐based enrichment, the amplification‐based INEAST bioassay expanded the dynamic range by two orders of magnitude toward sub picomolar concentration level. In particular, the 10 pm *TP53* cfDNA targets caused significant phase responses by 0.55 radian upon INEAST amplification (Figure S5k–i, Supporting Information), which was ≈7.6 folds higher than the responses elicited by the interfacial cfDNA hybridization. Based on the real‐time biosensing results, the nonlinear regressions with Hill equation were established toward the four targeted cfDNA sequences (Figure 3g; Figure S5j,i,n,p, Supporting Information). The LoD of the proposed INEAST bioassay for the four cfDNA targets, i.e., *PTEN*, *EGFR*, *CDKN2A*, and *TP53* were found to be 0.02 pm (12 copies per µL), 0.04 pm (24 copies per µL), 0.01 pm (6 copies per µL), 0.04 pm (24 copies per µL) respectively, which were ≈1000 times more sensitive than the hybridization‐based enrichment LSPR assays (Figure 3h and Table S4, Supporting Information). Our investigation into various cfDNA detection techniques and the clinical cut‐off values for cfDNA bioassays revealed that the LoD and dynamic ranges of the INEAST biosensing system satisfy the requirements for clinical LC testing (Tables S5–S7, Supporting Information). Therefore, the established regression curves for the four cfDNA targets were utilized for quantitative detection of clinical patient plasma samples for cancer diagnosis.

### 2.4 Thermoplasmonic‐Enhanced cfDNA Specificity Using the INEAST Platform

**Figure 4:**
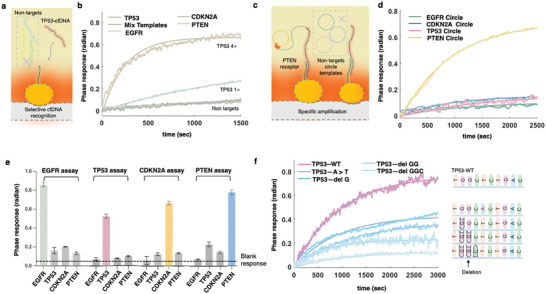
Sensitivity and selectivity of the detection platforms. a) Schematic diagram of the thermoplasmonic enhanced INEAST bioassay that identify non‐targeted and mismatched cfDNA. b) Real‐time sensorgrams of the INEAST *TP53* bioassays for recognizing the specific and non‐specific cfDNA targets. c) Schematic illustration of the thermoplasmonic enhanced INEAST bioassays that identify circle template mismatches for selective amplification. d) Real‐time sensorgrams of the INEAST *PTEN*‐amplification bioassays for recognizing the specific and non‐specific circular DNA template. e) Selectivity of the four INEAST bioassays for quantitative identifying the designated cfDNA target. Bar‐plot demonstrates INEAST phase response toward targeted or non‐targeted cfDNA. f) The sensorgrams of the INEAST *TP53* bioassay for characterizing the biosensing responses toward mutations in *TP53* cfDNA sequences. Four mutations, including A‐T variant, G deletion, GG deletion, and GGT deletion, were compared with the wide‐type *TP53* cfDNA.

Conventional RCA amplification at room temperature may face challenges such as non‐specific hybridization and false amplification. In contrast, the INEAST hybridization and amplification processes regulated by the thermoplasmonic effect can demonstrate higher specificity in terms of selective cfDNA recognition and sequence amplification. Therefore, we further validated the specificity performance of the developed INEAST cfDNA bioassays by testing non‐targeted and mismatched sequences. To evaluate the INEAST selectivity toward non‐target sequences, the amplification‐based biosensing performance toward *TP53* cfDNA targets was characterized by adding multiple interfering cfDNA sequences, i.e.*, EGFR*, *CDKN2A*, *PTEN* (**Figure** **4**a). The specific *TP53* targets with 50 pm concentrations produced a strong phase response of 0.64 radian. In contrast, the specimens only containing non‐targeted cfDNAs such as *EGFR*, *CDKN2A*, and *PTEN* were unable to initiate the INEAST amplification processes and produced a much‐diminished signal at ≈0.09 radian (Figure 4b). Moreover, when analyzing an equimolar mixture of 12.5 pm *EGFR*, *TP53*, *CDKN2A*, and *PTEN*, only the specific *TP53* targets yielded an effective phase response of 0.25 radian, as shown in Figure 4b. These results indicated the high specificity of the proposed INEAST bioassay.

Similarly, the specificity of the circular DNA template was further verified as shown in Figure 4c–e. Taking the *PTEN* cfDNA bioassay as an instance, the amplification process within the INEAST bioassay was activated and produced a robust phase change of 0.67 radians when *PTEN*‐cfDNA were selectively bonded with the designated circular templates. In contrast, non‐specific circular‐DNA templates for *EGFR*, *CDKN2A*, and *TP53* detection only produced diminished phase responses below 0.2 radians (Figure 4e; Figure S6a–c, Supporting Information).

In addition, the capability of INEAST to recognize defined mismatches was subsequently investigated by introducing designated cfDNA site mutations (Figure 4f). The single A‐T mismatch within the *TP53*‐cfDNA sequence was recognized with the INEAST biosensing system and yielded a 0.4‐radian amplification response as shown in Figure 4f. In contrast, the GC deletion, which had a slightly higher impact on the circular‐DNA hybridization, showed lower levels of amplification signal at 0.33 radian. By further increasing the number of mismatches, the introduced double mismatches and triple mismatches, i.e., GG deletion and GGC deletion within the *TP53*‐cfDNAs, caused further suppression of the INEAST responses by reaching only 0.22 and 0.12 radian respectively. The INEAST biosensing responses caused by the triple base‐pair mismatches were comparable to the blank measurement (Figure 4f), demonstrating that the INEAST bioassay is able to fully discriminate cfDNA sequences with more than two base‐pair mismatches. It was worth noting that the declined INEAST amplification bioassay responses were consistent with the elevated impact toward the melting temperature of the binding domain within the circular sequences. These results suggested that an optimized interfacial thermoplasmonic heating facilitated the discrimination of cfDNA mutations with excellent specificity for analyzing nucleotide polymorphisms. Despite single‐site mutations still triggering weak biosensing signals, differential analysis of the INEAST phase responses can facilitate identifying cfDNA sequence alterations (Figure 4f; Figure S6a–c, Supporting Information).

Validation experiments with real patient samples further verified the specificity and accuracy of the INEAST biosensing system. Compared with the standard detection of different concentrations at 1 nm, 100 pm, and 10 pm, the calculated recovery based on the INEAST LSPR biosensors were determined to be 108.7%, 99%, 103% in the clinical patient samples respectively (Table S8, Supporting Information). These experimental results further demonstrated that the INEAST LSPR system, enabled by in situ thermoplasmonic regulation, can specifically detect target cfDNA sequences with high quantification accuracy.

### 2.5 INEAST Characterization of Plasma cfDNA Expressions in Lung Cancer

**Figure 5:**
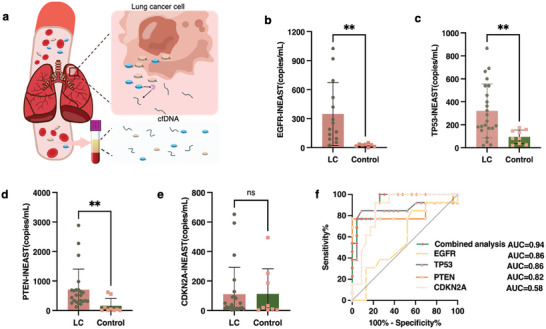
INEAST‐based cfDNA detection performance for clinical cfDNA liquid biopsy and LC diagnosis. a) Schematic diagram of the production and cfDNA collection process in LC patients. b–d) The INEAST‐based cfDNA quantification of the (b) *EGFR*, (c) *TP53*, (d) *CDKN2A*, and (e) *PTEN* expression levels in lung cancer patients and control group. The box plot presents the median, inter‐quartile range, min–max range. Bars represent standard errors. f) ROC curves demonstrated the INEAST‐based lung cancer diagnosis performance with the different cfDNA biomarkers.

The utilities of the INEAST biosensors for LC diagnosis were examined by quantifying multiple cfDNA expression levels from clinical patient plasma samples. In this work, a total of 36 clinical participants, including 23 active LC patients and 13 control cases, were studied. The targeted individuals were selected based on systematic criteria, which indicated that the LC and control patients demonstrated no significant difference in the aspects of gender, age, smoking, and medical intervention history (Table S9, Supporting Information). The 13 control individuals represented patients who were at high risk but had not been found to have malignant lung cancer. As shown in **Figure** **5**a, the serum samples collected from LC patients were pretreated to extract nucleic acid with Mag‐Bind paramagnetic particles for downstream INEAST analysis. The sequence information of the circulating cfDNA was initially analyzed, and the expression levels of the four different cfDNA targets in the blood samples of the 23 active LC patients and the 13 control individuals were provided in Table S10 (Supporting Information).

Based on the INEAST biosensing results toward the four cfDNA biomarkers as shown in Figure 5b–e, we confirmed that the overall expression levels of *EGFR*, *TP53*, and *PTEN* were higher in the lung cancer group compared to the control group as shown in Figure 5b–d. The *CDKN2A* showed diminished significant differences between the two groups (Figure 5e). Concurrently, the quantitative PCR was applied to analyze the same extracted cfDNA samples as shown in Figure S7a–d (Supporting Information). The developed INEAST bioassay showed an excellent correlation with the qPCR results as shown in Figure S7e–h (Supporting Information). Subsequently, the optimal cut‐off value for distinguishing between tumor and control samples was calculated using a receiver operating characteristic (ROC) analysis for each cfDNA. By applying the optimal cut‐off value for each individual cfDNA biomarker, the observed sensitivity and specificity for LC diagnosis ranged between 51.7% to 77.8% and 72.0% to 88.0% respectively (Table S11, Supporting Information). Among them, *TP53* showed one of the best diagnostic efficacies by achieving an AUC of 0.86 with 91.30% specificity and 84.62% sensitivity. *CDKN2A* only showed diminished diagnostic efficacy with an AUC of 0.58. The combined analysis of the four cfDNA bioassays achieved significantly improved diagnostic performance with an AUC of 0.94, which further demonstrated the primary benefit for increased accuracy in lung cancer diagnosis, as shown in Figure 5f. Following this, the gold standard PCR was employed to demonstrate the detection utility of INEAST bioassay for clinical samples. Taking *TP53* as an example, the diagnosis efficacies achieved an AUC of 0.85 with 84.62% specificity and 95.65% sensitivity (Figure S8a, Supporting Information). Notably, the combined analysis of PCR‐based bioassays also achieved an improved diagnostic performance with an AUC of 0.95, which demonstrated the competitive diagnostic performance with the INEAST bioassay.

These clinical testing results collectively demonstrated that the multiplexing INEAST bioassays enabled straightforward, rapid, and label‐free detection of cfDNA in clinical samples. This plasmonic liquid biopsy technique with high sensitivity and specificity has the potential to provide valuable support for clinical cancer diagnosis.

### 2.6 Clinical Validation via Profiling Biomarkers in Blood Sample for Lung Cancer Classification

**Figure 6:**
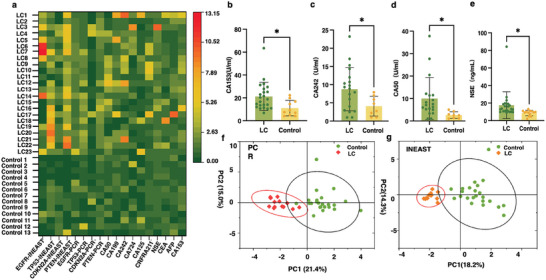
The INEAST bioassays for lung cancer classification. a) Heat map representation of the cfDNA and glycosylation biomarkers for LC classification. b–d) The quantitative analysis of the expression levels of glycosylation protein biomarkers (b) CA15‐3, (c) CA242, (d) CA40, and (e) NSE in the lung cancer patients and the control group. f) A PCA‐based tumor diagnostic model that took the PCR‐determined cfDNA expression levels and the glycosylation biomarker expression levels into account. g) A PCA‐based tumor diagnostic model that took the IEAST‐determined cfDNA expression levels and the glycosylation biomarker expression levels into account.

The glycosylation biomarkers that frequently found in the early stages of tumor development have been clinically used as conventional tumor biomarkers for diagnostics and prognosis.^[^ ^19^ ^]^ As cancerous hallmarks, glycosylation biomarkers, i.e., carbohydrate antigen CA19‐9, CA125, and CA15‐3, were aberrantly expressed in cancer and play significant roles in tumor growth and metastasis.^[^ ^20^ ^]^ Diagnostic models combining blood glycosylation patterns and nucleic acid biomarkers like cfDNAs have the potential to more precisely reflect LC progression for cancer diagnosis and prognosis.^[^ ^21^ ^]^ To further demonstrate the superiority of the INEAST‐based liquid biopsy and LC plasma diagnosis, the cfDNA levels retrieved by INEAST bioassays and clinical glycosylation biomarkers were harnessed to discriminate between LC patients and control individuals, as shown in **Figure** **6**a and Table S12 (Supporting Information). The bioassay results, including the INEAST‐retrieved cfDNAs, PCR‐retrieved cfDNAs, and ten different glycosylation biomarkers, were normalized based on the mean concentrations of the investigated tumor biomarkers in the control group (*n* = 13). The differences in the cfDNA expression between the lung cancer group (*n* = 23) and the control group (*n* = 13) were subsequently analyzed.

As shown in Figure 6b–e and Figure S9a–f (Supporting Information), glycosylation‐associated tumor biomarkers like CA15‐3, CA242, CA50, NSE, CA19‐9, and CYRA211 exhibited diminished differences (*p*  ≤  0.05) in distinguishing the LC group from the control group. However, the ROC curves indicated that single glycomarker demonstrated limited AUC level (Figure S8, Supporting Information). Moreover, non‐cancer control donors and LC patient groups manifested no significant differences in a number of glycosylation biomarkers including CEA, AFP, CA724, and CA125 (Figure S9a–f, Supporting Information). Therefore, we considered the combination of clinical glycosylation biomarkers and the proposed cfDNA biomarkers to establish a reliable model for early lung cancer diagnosis. As illustrated in Figure S8b (Supporting Information), the ROC analysis of the combinational bioassay, which incorporated both glycosylation and cfDNA biomarkers, attained AUC value of 1.0 with the INEAST‐based cfDNA bioassay. Moreover, the combinational bioassay using PCR‐based cfDNA results also achieved an improved AUC of 0.96. These findings further demonstrated that the accuracy of lung cancer diagnosis can be enhanced by the combined diagnosis of multiple cfDNA and glycosylation biomarkers.

The classification results by considering both glycosylation and cfDNA biomarkers were illustrated in Figure 6f,g. The high‐dimensional cfDNAs and glycosylation‐based diagnostic results were simplified through principle component analysis (PCA), and the two dominated principle components (PCs) captured 18.2% and 14.3% contributions as shown in Figure 6g. Accordingly, the INEAST bioassays achieved reliable LC diagnosis in distinguishing healthy populations and LC patients when combined with clinical glycosylation metrics (Figure 6g). Meanwhile, the control features detected by the proposed INEAST assays demonstrated similar features and performance compared to the gold standard PCR, with 21.4% captured by PC1 and 15.0% captured by PC2 (Figure 6f). In summary, our results highlighted the value of the INEAST‐based liquid biopsy approach for cancerous tumor diagnosis. By cooperating with the established glycosylation bioassays, the proposed INEAST approach has great potential for implementation in clinical LC screening programs and achieves improved early‐stage diagnostic performance.

## 3 Discussion

Liquid biopsy with cfDNA bioassay represents a promising approach for LC diagnosis and prognosis. To address these needs, we have developed a multifunctional INEAST biosensing system that combined interfacial thermoplasmonic effects with LSPR to amplify and highly sensitive detect cfDNA for LC liquid biopsies. Using the 2D plasmonic nano‐absorber, the AuNI plasmonic chip has been proven to manipulate local PPT heating and facilitate in situ hybridization and amplification to achieve highly specific, sensitive, and precise cfDNA quantification. In addition, multiplexing detection of cancer‐associated cfDNA biomarkers, including *EGFR*, *TP53*, *CDKN2A*, and *PTEN* has enabled a noninvasive and cost‐effective cancer diagnosis method for differentiating lung cancer cases.

The INEAST bioassay, which harnessing the interfacial amplification process on plasmonic chips, demonstrated a stable and straightforward isothermal technique for cfDNA amplification.^[^ ^22^ ^]^ Compared to the conventional NAAT approaches, INEAST achieved label‐free and real‐time cfDNA detection by employing the interfacial LSPR effects. In term of biosensing performance, the on‐chip and interfacial plasmonic bioassays significantly improved the reliability and applicability for quantitative cfDNA analysis. First, the cfDNA enrichment and amplification processes can be simultaneously performed within the same plasmonic biosensing interface, thereby eliminating the need for additional interventions compared to the conventional bulk liquid‐phase NAAT assays. Second, the direct cfDNA hybridization and amplification within the plasmonic near‐field enabled a label‐free biosensing manner and outperformed the conventional fluorescent readout approaches in terms of bioassay sensitivity and cost‐effectiveness.^[^ ^23^ ^]^ The MFP LSPR sensing scheme exhibited femtomolar‐level LoDs for detecting the four cfDNA targets. Moreover, the INEAST bioassays demonstrated superior dynamic ranges for clinical application. For instance, the *EGFR* amplification bioassay yielded a dynamic range for *EGFR* cfDNA amplification of 0.04 to 10 000 pM, while the linear dynamic range was established between 35 and 7000 pm (Figure S10 and Table S13, Supporting Information).

Moreover, interfacial thermoplasmonic effect also significantly improved the bioassay kinetics and potentially diminished false positive results for clinical diagnosis. In conventional RCA bioassays, molecular interaction and biochemical reactions conducted at room temperature hinder the maintenance of a rigid structure of the nucleic acid strand, thereby presenting challenges for efficient nucleic acid hybridization and amplification.^[^ ^24^ ^]^ The length of the nucleic acid sequence correlates with increased complexity of the secondary structure, which adversely affects the performance of hybridization and amplification bioassays. For instance, the folding structures of the *TP53* cfDNA sequences of varying lengths were predicted as shown in Figure S11 (Supporting Information). The long cfDNA sequences with 166 nt in length exhibited complex binding structure and high Gibbs energy, suggesting poor rigidity for biosensing. It is known that the DNA conformation, rigidity, and internal strand structure can be altered by manipulating temperature, electrostatics, π interactions, and hydrophobicity. Therefore, effective thermoplasmonic temperature control plays a vital role to improve the hybridization and amplification kinetics for highly sensitive cfDNA detection. Figure 2f also demonstrated that the cfDNA amplification efficiency was significantly enhanced 5.18, 7.33, and 9.25 times by employing the photothermal‐enhanced INEAST bioassays. Additionally, the INEAST bioassays achieved NAAT within 40 min, whereas the liquid‐phase RCA generally required more than 2 h, Table S7 (Supporting Information). The improved amplification efficiency with the INEAST temperature regulation is even more critical for quantifying trace amount of cfDNA targets.^[^ ^25^ ^]^

In terms of clinical applicability, we also demonstrated that the INEAST bioassay may facilitate the development of novel liquid biopsy for early cancer diagnosis and disease management. Specifically, the high sensitivity, short assay time, and flexible detection manner make the INEAST bioassay a strong candidate for point‐of‐care LC testing. Considering the short cfDNA half‐life, the short assay time (<40 min) significantly benefits accurate quantification of circulating cfDNA, which diminishes the false negative results in screening LC cases. Additionally, the high sensitivity also indicated that the INEAST bioassay required less volume of blood samples for diagnosis. This study attempted to establish a cfDNA‐based risk prediction model for lung cancer diagnosis. Despite only four cfDNAs being considered, the INEAST‐based model exhibited satisfactory performance in the classification of malignant and control cases. In addition, by combining with existing clinical glycomarker assays, the INEAST biosensing approaches exhibited the potential to be considered as a comprehensive paradigm for more precise cancer diagnosis. These advances render INEAST a superior non‐invasive, cost‐effective, and routine screening strategy for detecting early‐stage cancer in high‐risk populations.

While this study demonstrated a proof‐of‐concept early diagnostic biosensor that potentially used for cfDNA‐based lung cancer screening and risk assessment, it is noticeable that more extensive validation of the INEAST‐based LC risk prediction model is demanded. This should involve larger sample sizes, which also allow stratified analyses to assess how well the proposed cfDNA biomarkers can predict lung cancer cases associated with different characteristics, such as the LC stages and histological subtypes. We also acknowledged that the long‐term follow‐up diagnosis of participants is an effective approach for validating the applicability and reliability of the INEAST biosensing platform in detecting early‐stage tumors. The Long‐term follow‐up potentially allows us to assess whether the benefits observed shortly after screening persist over time.

In summary, we have developed a label‐free INEAST liquid biopsy assay for high sensitivity and specificity cfDNA quantification and cancer diagnosis. By harnessing the thermoplasmonics and LSPR near‐fields, the proposed INEAST bioassay achieved highly efficient cfDNA enrichment, amplification, and label‐free transduction on the solid‐phase plasmonic chip. The INEAST bioassay demonstrated excellent sensitivities, and the LoDs toward *PTEN*, *EGFR*, *CDKN2A*, and *TP53* were found to be 0.02, 0.04, 0.01, and 0.04 pM, respectively. Additionally, our INEAST bioassay does not require sophisticated temperature control instruments and professional operators. In terms of clinical applications, this work lays the groundwork for developing an inexpensive test to measure multiple cfDNA biomarkers in patient plasma that can distinguish between lung cancer and non‐cancer donors. Especially when incorporated with other protein‐based assays, this novel amplification‐based biosensor exhibits promise for potential clinical utilities in early‐stage cancer diagnosis.

## 4 Experimental Section

### Amplification Reagents and DNA Templates for INEAST

The nuclear acid sequences were purchased from Genescrip (Nanjing, China). The T4 DNA ligase and exonucleases I and III were purchased from NEB (New England Biolabs). The circle template solution buffer was prepared by mixing 1 µm Padlock agent and 3 µm assistant sequences. The prepared solution was initially heated at 65 °C for 10 min, subsequently cooled down to room temperature for 20 min. In addition, 10× T4 DNA ligase reaction buffer and T4 DNA ligase were added to the above solution at 16 °C for 5 h, the reaction system was incubated to complete the intramolecular ligase of the padlock. Finally, exonucleases I and III were added to cut off the excess nucleotides and finalized the production of the circulate templates. The INEAST working liquid buffers were prepared by mixing 0.5 U uL^−1^ phi29 DNA polymerase, 250 µm dNTP mixture, 50 nm circular DNA templates (Sequence information in Table S1, Supporting Information), and 50 nm cfDNA capture sequence.

### The Preparation of AuNI Chip

Gold nanoislands (AuNIs), i.e., the INEAST biosensor chips were synthesized by using a thermal annealing method. In detail, a gold nanofilm with a nominal thickness of 5.0 nm was deposited on a clean BK7 glass substrate through magnetron sputtering (Denton Vacuum). The sputtered Au nanofilms were thermally annealed at 550 °C for 3 h to produced densely distributed AuNIs with a nominal diameter of 40 nm on the glass surface. By regulating the thickness of the gold nanofilm, the plasmonic spectrum and peak absorption wavelength of the AuNI chip can be tuned. The visible light absorption of each AuNI chip was tested to retrieve the ideal plasmonic resonance conditions.

### Phase‐Sensitive Interferometric LSPR Biosensors Within INEAST System

In the interferometric LSPR phase sensing system, the wide‐spectral white light beam was generated via an LED source and subsequently linearly polarized by a polarizer (P1). One birefringent crystal BC with sufficient retardation was utilized to construct a spectral interferogram for phase‐sensitive LSPR transduction. Afterward, the modulated incident light was coupled into the AuNI−dielectric interface at an inclined incident angle of 72°. The interferometric spectra were finally recorded by the fiber‐coupled spectrometer (AvaSpec, Avantes). In addition, a homogenized 532 nm laser beam was irradiated for generating thermoplasmonic heating at the normal incident angle. The local thermoplasmonic heating temperature on the AuNI biosensing interface can be optimized by fine‐tuning the irradiation laser power density.

### INEAST Working Flow

In the functionalization process, 300 µL of 0.15 nm cDNA capture sequences were introduced into the AuNI detection chamber for 30 min. Following buffer flushing with PBS solution, the blank sites of the AuNI chips were further blocked for 15 min using MCH diluted in absolute ethyl alcohol. The LSPR biosensing system monitored the Au–S functionalization and blocking processes in a real‐time manner. The functionalized AuNI chips were subsequently used to capture and enrich the cfDNA targets. The designated laser power was applied onto the AuNI sensor chip for thermoplasmonic regulation. The long‐pass filter with a cut‐on wavelength at 552 nm was utilized to diminish the 532 nm laser impact on the stability of the LSPR transduction. By adding the prepared amplification reagents, the on‐chip amplification process was initiated. The corresponding phase responses were retrieved from the phase sensorgrams during the whole process of INEAST biosensing.

### Gel Electrophoresis

The mixture containing 10 µL of 10× Gel sample loading buffer and 1.5 µL of target sample was loaded onto a 1% agarose gel that has been stained with SYBR DNA gel stain (BioAssay Co., Ltd.). The electrophoresis was performed with a Mudid‐2Plus device from Taraka Bio Inc. A DNA molecular marker of 100 bp DNA ladder (Takara Bio Inc) was incorporated to assess the size of the amplicon byproducts. For characterizing the nucleic acid amplification products, a total of 15 µL reaction solutions were prepared by mixing cfDNA, dNTPs, cDNA circulate template, and phi 29 DNA polymerase. The amplification reaction was continued for 1 h at 30 °C. After incubation, each reaction solution was resolved on a 2% polyacrylamide gel using 1 × TBE as the running buffer. Gel electrophoresis was conducted at a constant voltage of 125 V for 50 min, and finally imaged by the ChemiDoc Imaging System (Bio‐Rad).

### Study Design and Participants

The use of INEAST biosensors was assessed in a prospective cohort study done in oncology and outpatient clinics at Huadong Hospital Affiliated to Fudan University, with ethical approval from the Ethics Committee of Huadong Hospital Affiliated to Fudan University (No.20240093). Participants underwent diagnostic assessments for suspected cancer and have no cancer‐related medical intervention and treatment were considered in this study. The inclusion criteria for the LC group are as follows: 1) No history of malignant tumors, and no prior surgical or chemotherapy‐related treatments. 2) Biopsy and pathological confirmation according to the WHO classification of lung tumors. High‐risk asymptomatic subjects, i.e., the control group, are included under the following conditions: 1) Selection of patients with benign lung tumors, benign lung diseases, or pulmonary shadows. 2) Cases in this study will be matched with a control group based on age at recruitment, blood draw time, and gender. Exclusion criteria include: 1) Subjects under the age of 18; 2) Subjects with a history of tumors; 3) Participants who have received chemotherapy (including anticancer drugs), immunotherapy, hormone therapy, or radiation therapy. A central or local institutional review board approved all study procedures.

### Blood Sample Collection and cfDNA Extraction

Peripheral blood samples from included investigators were collected in EDTA collecting vessels. The plasma was isolated via 3000×g for 10 min. cfDNA in plasma were extracted from 2 mL of plasma from each sample with OMEGA cfDNA kit (Mag‐Bind cfDNA Kit M3298, Guangzhou, China), following the manufacturer's instructions.

### Quantitative PCR (qPCR) Analysis

Quantitative PCR was carried out with TB Green Premix Ex Taq (TAKARA, biomedical technology, Beijing, China) using Roche Light Cycler 96. The primer sequences are listed in Table S2 (Supporting Information). The cfDNAs were isolated from 2 mL blood plasma of LC patients or control individuals by cfDNA kit and quantitated via NanoDrop One spectrophotometer (Thermofisher, USA). To prepare the PCR reaction solution, 12.5 µL of SYBR Green mix, 4 µL of template cfDNA, 1 µL of forward primer sequence, 1 µL of reverse primer sequence, and 6.5 µL of nuclease‐free water were mixed together. In contrast, the negative control was made with the same PCR mixing buffer, but 4 µL of nuclease‐free water was used instead of the target cfDNA template. The qPCR analysis was initiated at 95 °C for 15 min, followed by 40 thermocycles consists of denaturation at 95 °C for 30 s, annealing at 60 °C for 30 s, and extension at 72 °C for 30 s on a real‐time PCR instrument (BioRad). All sample testing was repeated three times in three tubes, and the number of cycles experienced when the fluorescence signal reached the threshold in each reaction tube was recorded for quantitative analysis.

### INEAST Biosensing Characterizations

Linearity across the analytical measurement variety (*EGFR*, *TP53*, *PTEN*, *CDKN2A*, 10 nm‐500 fM) was assayed via applying the calibrators for three replicates at each concentration. The lower LoQ, demarcated as the lowest concentration that provides a signal‐to‐noise ratio of >10, and the LoD represents the lowest concentration that products a signal‐to‐noise ratio of >3. Recovery was evaluated by a known amount of analyte (*TP53*,1 nm, 100 pm, 10 pm) into the mix nucleic acid samples which were extracted from human plasma. All measurements were performed three times, and the recovery was calculated as [(final concentration − initial concentration)/added concentration]. In the mutation detection with the INEAST biosensors, four *TP53* cfDNA variants, i.e., A‐T mismatch, GC deletion, GG deletion, and GGC deletion were analyzed. The efficiency of identifying mutation was calculated as the proportion of the WT *TP53* bioassay result.

### Statistical Analysis

Statistical analyses were conducted using the MATLAB v2023b. The statistic significances were calculated by GraphPad Prism version 9.5.1 and all the data were shown as mean ± s.d. The two‐tailed Student's *t*‐test was used to compare differences between two groups with a *p*‐value < 0.05 as a threshold for significance. Hill equation was applied to description the calibration curves for INEAST bioassays^[^ ^26^ ^]^

(1) $$R=R_{lim}\;\times {\left(C/C_{0.5}\right)}^{h}/\left(1+{\left(C/C_{0.5}\;\right)}^{h}\right)$$

In this work, *R* was the biosensor response (signal), *C* referred to the concentration of analyzed cfDNA substance, *R_lim_* referred to the limiting value of *R* at C → ∞ (*R_lim_*), *C_0.5_* referred to the concentration of half saturation. The calculated Hill equation parameters of the INEAST bioassays are shown in Table S4 (Supporting Information).

## Conflict of Interest

The authors declare no conflict of interest.

## Supporting information



Supporting Information

## Data Availability

The data that support the findings of this study are available from the corresponding author upon reasonable request.
