# Supplementary material for: Interfacial cfDNA Enrichment and Amplification with On‐Chip Thermoplasmonics for Highly Sensitive Cancerous Liquid Biopsy
Source: Adv Sci (Weinh). 2024 Dec 4;12(4):2409708. doi: 10.1002/advs.202409708 (PMC11789577; doi:10.1002/advs.202409708)
Supplement: Supplementary file 1 — Supporting Information [file ADVS-12-2409708-s001.pdf]

ADVANCED SCIENCE
Open Access

Supporting Information

for *Adv. Sci.*, DOI 10.1002/advs.202409708

Interfacial cfDNA Enrichment and Amplification with On-Chip Thermoplasmonics for Highly Sensitive Cancerous Liquid Biopsy

*Danhua Wang, Linlin Liu, Wenjing Chi, Zhenping Liu, Jiayun Wu, Yirou Liang, Fei He, Ruixiang Zhang, Pengxin Huang, Yunbo Li and Guangyu Qiu**

Supplementary Information

Interfacial cfDNA Enrichment and Amplification with On-Chip Thermoplasmonics for Highly Sensitive Cancerous Liquid Biopsy

Danhua Wang[1], Linlin Liu[1], Wenjing Chi[2], Zhenping Liu[3], Jiayun Wu[1], Yirou Liang[1], Fei He[1], Ruixiang Zhang[1], Pengxin Huang[1], Yunbo Li[1], Guangyu Qiu[1]*

[1]Institute of Medical Robotics, School of Biomedical Engineering, Shanghai Jiao Tong University, Shanghai 200240, China

[2]Department of Laboratory Medicine, Huadong Hospital Affiliated to Fudan University, Shanghai, China

[3]The First People's Hospital of Linping District, Hangzhou 311100, Zhejiang Province, China

Corresponding author. Email: guangyuqiu@sjtu.edu.cn

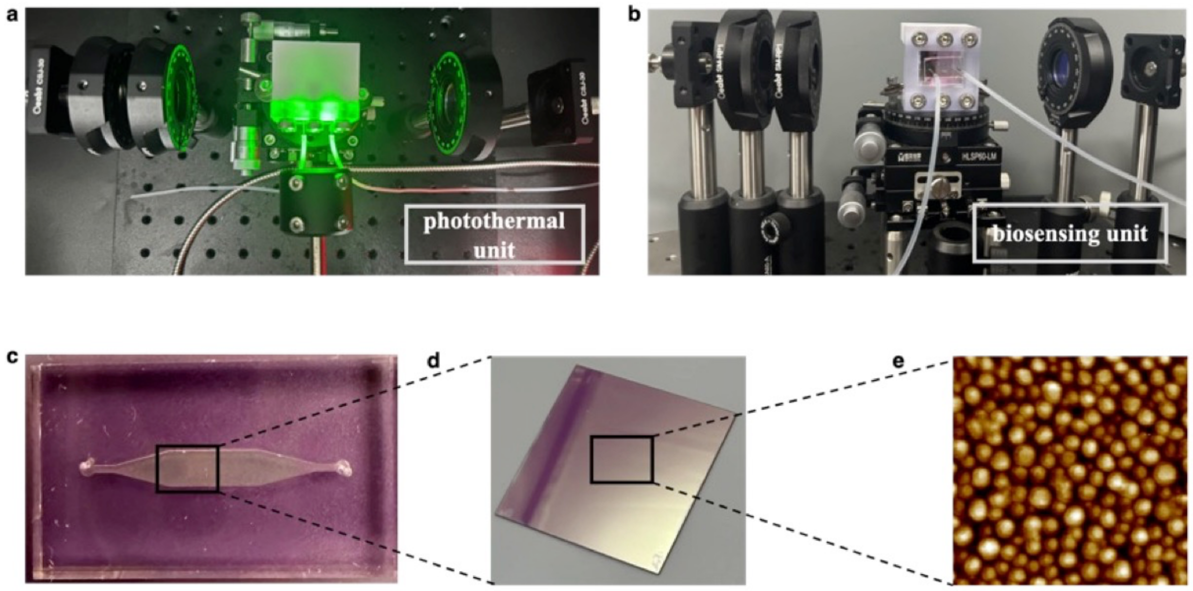

Supplementary Figure S1. The experimental setup and biosensor chips for the INEAST-based cfDNA bioassays. a. The system top-view image with the plasmonic photothermal unit for the temperature-regulated cfDNA biosensing; **b.** The optical system showed the common-path interferometric LSPR biosensing unit; **c**. The microfluidic-based AuNI biosensor chip; **d.** The AuNI biosensor chip as the INEAST transducing matrix; **e.** The AFM height-scanning image of the AuNI sensor chip; the image size is 500nm x 500nm, and the synthetic AuNI demonstrated a nominal diameter of 40nm.

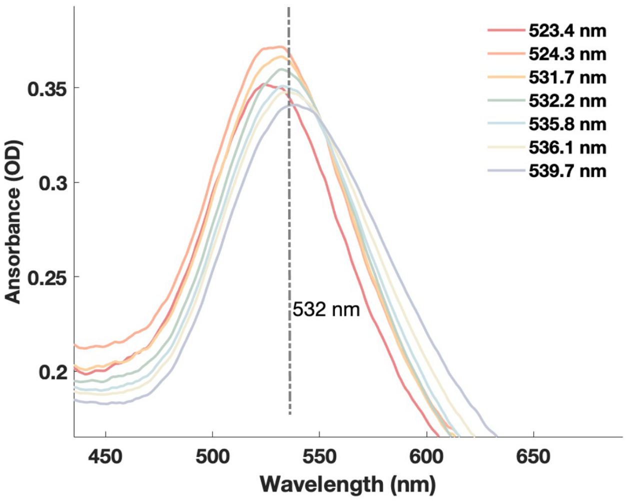

Supplementary Figure S2. The optimization of AuNI peak absorbance. The absorbances of the AuNI sensor chips were fine-tuned from 523.4 to 539.7 nm (±0.2 nm) by optimizing the thickness of thermal dewetted Au nanofilm.

The optimization of the AuNI chip involves adjusting its peak absorption wavelength to 532 nm by fine-tuning the thickness of thermos-dewetted gold nanofilm, which is crucial for improving the conversion efficiency in thermoplasmonic applications. The magnetron-sputtering technique was employed to deposit a thin layer of gold nanofilm onto a cleaned BK7 glass substrate. By adjusting the nominal thickness of the Au nanofilm, the plasmonic properties of the AuNI sensor chip can be regulated. Specifically, the thicker Au nanofilms generally exhibited demonstrated redshifted plasmonic peak absorptions due to an enlarged AuNI diameters and interparticle gaps compared to the thinner Au nanofilms. Therefore, optimization of the sputtering thickness allowed for tuning the absorption peaks to approaching the thermoplasmonic laser excitation wavelength of 532.2 nm (±0.2 nm) as demonstrated in the attached absorption spectra (**Figure S2, Supplementary Information**). By ensuring that the absorption peak closely matched the excitation wavelength of commonly used lasers (like those emitting at 532 nm), the conversion efficiency in thermoplasmonics can be significantly enhance. Notably, under ATR conditions with an incident angle of 72°, the plasmonic resonance wavelength used for LSPR sensing demonstrated a redshift to 580 nm.

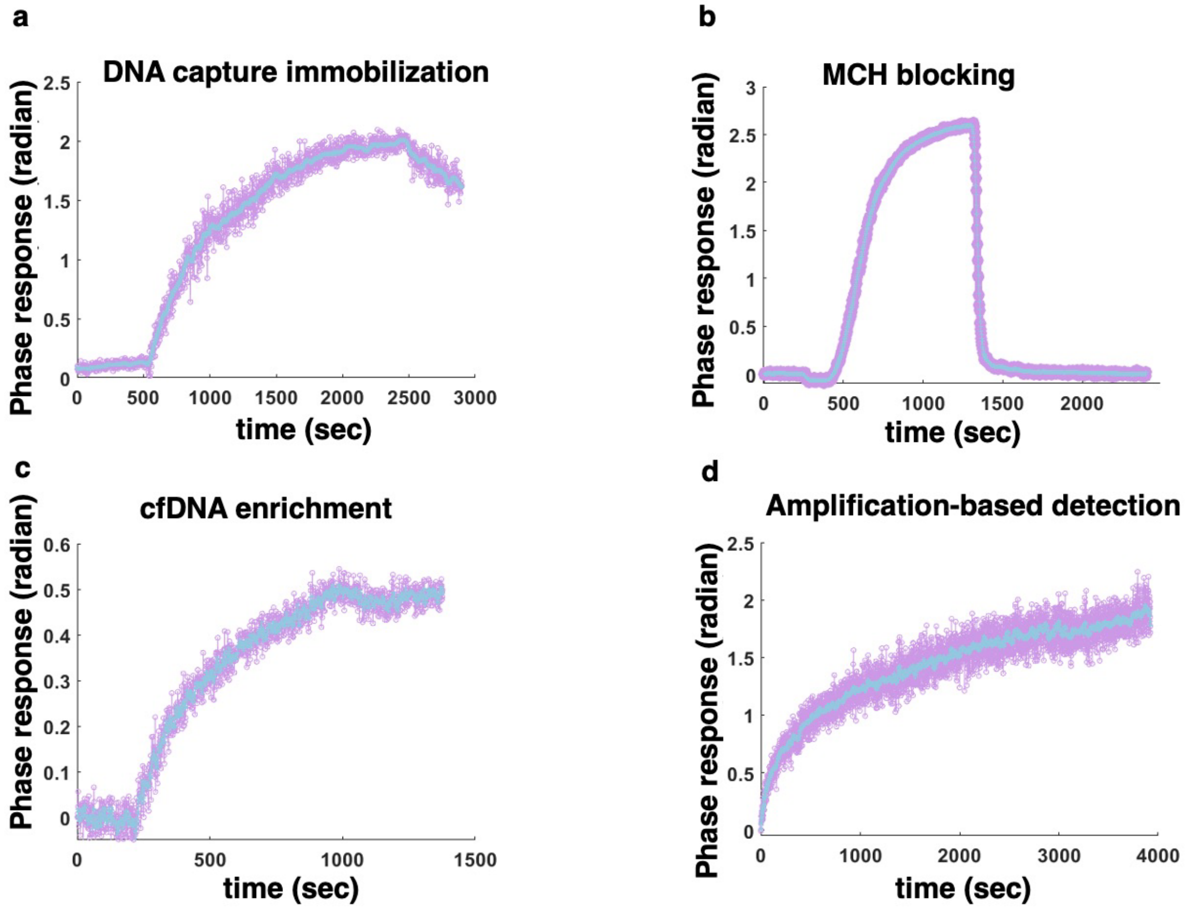

Supplementary Figure S3. Real-time monitoring of the AuNI functionalization, enrichment, and amplification processes for the INEAST-based cfDNA quantification. a. Real-time phase responses induced by the immobilization of cDNA captures; **b**. The plasmonic phase responses induced by the surface MCH blocking in the INEAST system for mitigating the non-specific binding events; **c**. Real-time phase responses produced by the specific cfDNA hybridization and interfacial enrichment; **d**. The phase responses of rolling cycle-based amplification within the INEAST interfacial biosensing system.

The surface functionalization of the AuNI sensor chips facilitated the INEAST biosensor to achieve highly specific cfDNA biosensing. Specifically, the thiolate cDNA capture sequences, *i.e.*, the *EGFR* cfDNA complementary sequences, were first covalently immobilized onto the AuNI photonic chips by forming Au−S bonds between cDNA-SH and AuNIs. This reaction caused a significant phase response by 1.9 radian, which indicated that the cDNA capture sequences were successfully combined with AuNI photonic chips. Then the MCH molecules were immobilized on the AuNI chips for biosensing surface blocking. This process triggered another significant phase response by 2.6 radian, since the dissolving ethanol buffer caused an enormous refractive index change within the LSPR system. When detecting the fully matched sequences, *i.e.*, the targeted cfDNA reached the functionalized biosensing surface and was selectively hybridized with the immobilized cDNA capture sequences. The molecular binding between cfDNA and cDNA receptor caused a localized refractive change and contributed a sharp plasmonic phase shift as shown in the **Figure S3c, Supplementary Information**. Upon the sequence-specific cfDNA enrichment, thermoplasmonics-enhanced on-chip INEAST reactions were developed for trace amount cfDNA quantification. Compared to hybridization detection, the amplification-based bioassay demonstrated higher sensitivity due to the elongation of the cfDNA chain. The amplification reaction initiated a phase response by 1.8 radian. Compared with the enrichment process, the amplification-induced LSPR phase responses were two times higher, which indicated that the cfDNA was amplified successfully. Additionally, the amplification-based INEAST bioassays demonstrated a high sensitivity due to the highly efficient interfacial amplification.

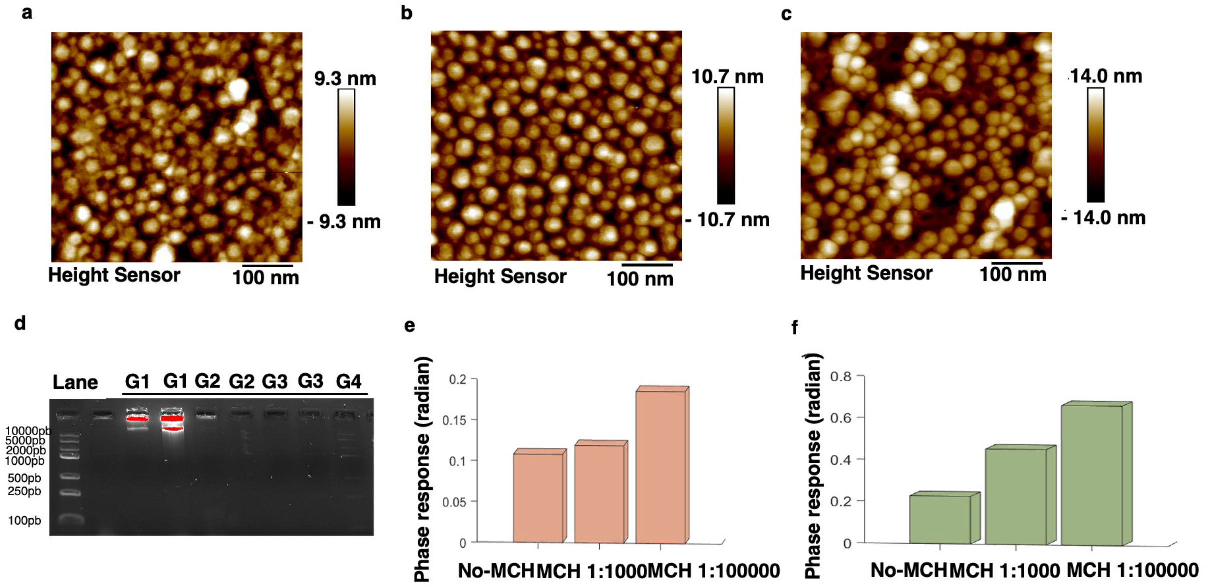

Supplementary Figure S4. Characterization of INEAST-based cfDNA amplification. a. The AFM height scanning map of the bared AuNI chip without any surface modification. The scale bar is 100 nm; **b.** The AFM height scanning map of the MCH-modified AuNI chip. The scale bar refers to 100 nm; **c.** The AFM phase map of the AuNI sensor after the interfacial rolling cycle cfDNA amplification. *TP53* cfDNA with 100 pM concentration was detected with the AuNI sensor chip; **d**. The gel electrophoresis was employed to verify the amplification products within different reaction systems; G1 groups, with all reactant presence; G2 groups, with only cfDNA template absence; G3 groups, with only phi29 polymerase absence; G4 groups, with only DNA circle absence; **e.** Phase responses of the *EGFR* enrichment bioassays demonstrated different LSPR phase responses due to the different MCH blocking conditions; **f.** The INEAST-based amplification bioassay produced different LSPR phase responses when using different MCH blocking conditions.

The concentration of MCH blocking agents was optimized as shown in **Figure S4e-f, Supplementary Information**. By considering the substantial impact on INEAST amplification efficiency, we ultimately opted to use MCH concentration of 0.1% for subsequent INEAST-based cfDNA bioassays.

The plasmonic enhanced photothermal effect have been characterized and the nucleic acid activities including DNA-RNA hybridization, enzyme-based amplification and dehybridization within the thermoplasmonic fields, which proved that the photothermal effect demonstrated diminished damages toward the nucleic acid structure [1]. One of the preliminary factors that guarantees the low damages toward biomolecules is the fine-tuned thermoplasmonic heating temperatures. The thermoplasmonic heating temperature utilized in this work was optimized to be 32.9 ^{o}C to eliminate potential negative impact to nucleic acid amplifications (**Figure 2**). The thermoplasmonic heating fields, constructed by a homogenized laser and designated power density can also mitigate the potential damages during the bioassay process. Moreover, the appropriate photothermal heating temperature can facilitate the hybridization of nucleic acids by maintaining their rigid structure of DNA sequences. Furthermore, the INEAST biosensing results, gel electrophoresis, and AFM results as shown in **Figure 2** and **Figure S4, Supplementary Information**, have verified that nucleic acids can be amplified within the INEAST biosensing system.

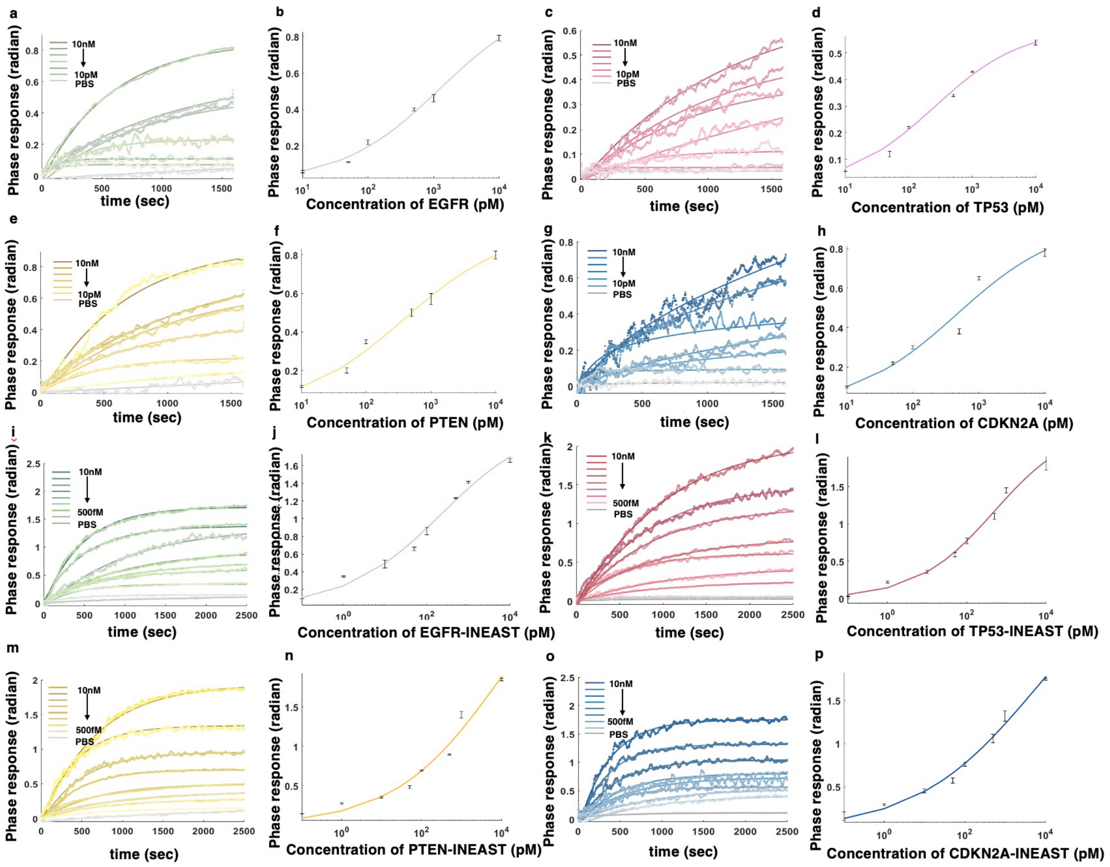

Supplementary Figure S5. Characterizations of quantitative biosensing performance of cfDNAs interfacial enrichment and amplification in the INEAST system. (**a**) Direct cfDNA hybridization within the INEAST biosensors were real-time monitored for *EGFR* cfDNA targets with (**b**) the Hill equation-based regression calibration curve; (**c**) Direct cfDNA hybridization within the INEAST biosensors were real-time monitored for *TP53* cfDNA targets with (**d**) the Hill equation-based regression calibration curve; (**e**) Direct cfDNA hybridization within the INEAST biosensors were real-time monitored for *PTEN* cfDNA targets with (**f**) the Hill equation-based regression calibration curve; (**g**) Direct cfDNA hybridization within the INEAST biosensors were real-time monitored for *CDKN2A* cfDNA targets with (**h**) the Hill equation-based regression calibration curve. Rolling cycle-based amplification processes with INEAST bioassays were real-time monitored for detecting (**i**) *EGFR* cfDNA targets and (**j**) the corresponding calibration curve; (**k**) *TP53* cfDNA targets and (**l**) the corresponding calibration

curve, (**m**) *PTEN* cfDNA targets and (**n**) the corresponding calibration curve; (**o**) *CDKN2A* cfDNA targets and (**p**) the corresponding calibration curve.

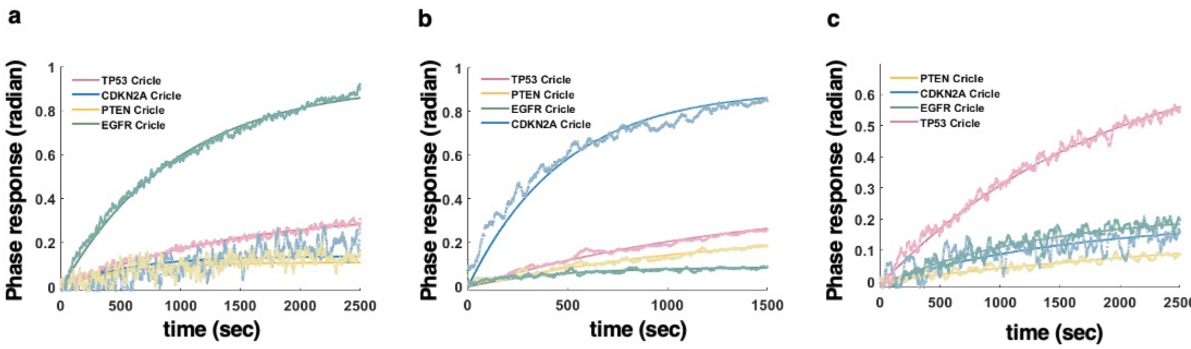

Supplementary Figure S6. The specificity characterization of the amplification DNA circles within the INEAST bioassays. a. The *EGFR* rolling DNA cycles were utilized to selectively amplify the target cfDNA sequence *EGFR* in mixed samples. The other three rolling DNA cycles produced diminished detection signals comparable to the blank measurement; **b.** The *CDKN2A* rolling DNA cycles were utilized to selectively amplify the target cfDNA sequence *CDKN2A* in mixed samples; **c.** The *TP53* rolling DNA cycles were utilized to selectively amplify the target cfDNA sequence *TP53* in mixed samples. The other three rolling DNA cycles produced diminished detection signals comparable to the blank measurement.

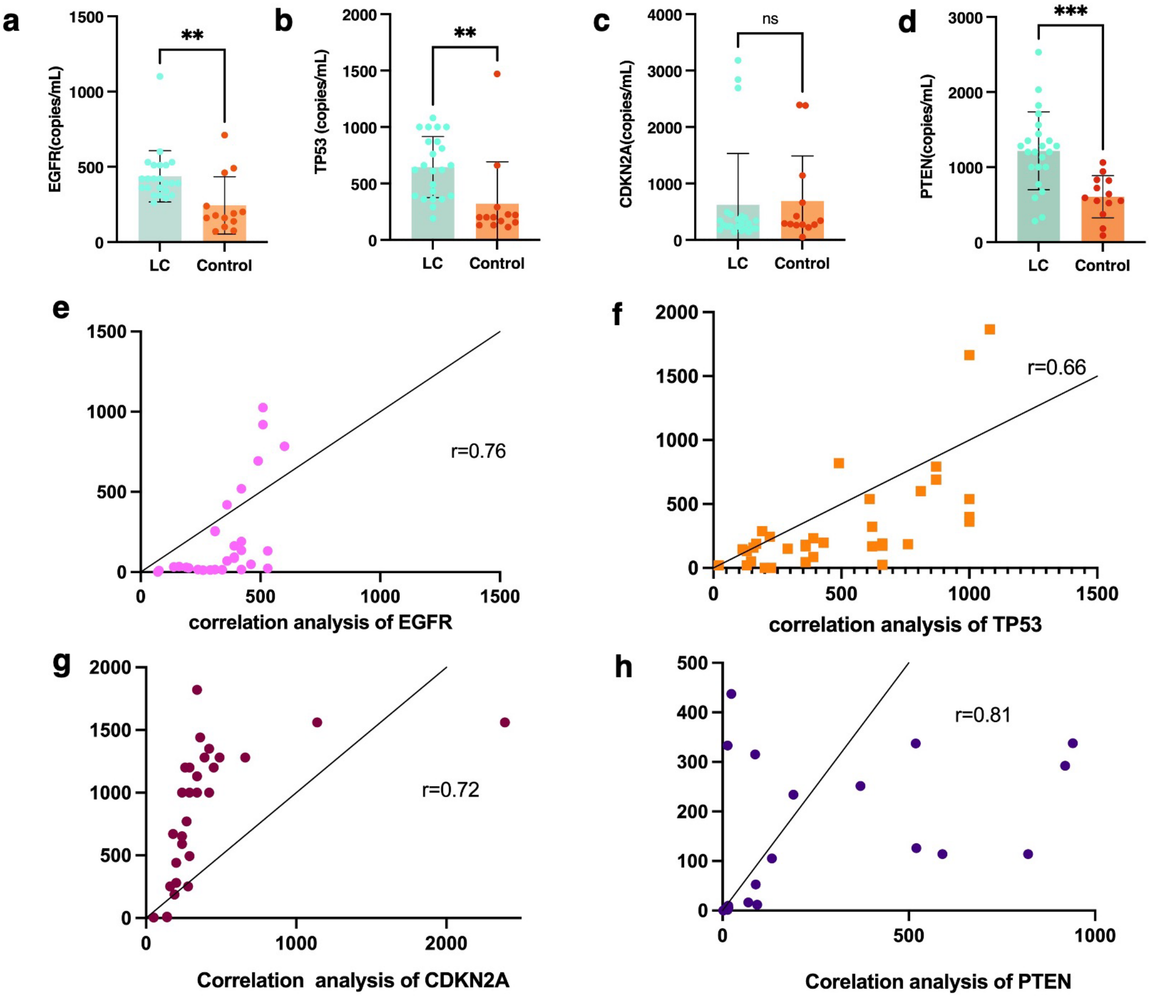

Supplementary Figure S7. The INEAST-based detection of four cfDNA expressions in clinical circulating blood samples. a-d, Comparison of the expression levels of the targeted four cfDNAs between LC patients and control groups. Histograms of relative expression levels for *EGFR* (**a**), *TP53* (**b**), *PTEN* (**c**), *CDKN2A* (**d**) for the lung cancer patients and the control group by qPCR. Correlation between expression levels measured by the gold standard qPCR approaches and the proposed INEAST method for the four cfDNA biomarkers, namely *EGFR* (**e**), *TP53* (**f**), *CDKN2A* (**g**), *PTEN* (**h**). Statistical analysis for *EGFR*, with Pearson r = 0.76 and $P \leq 0.001$, for *TP53* with r = 0.66 and $P \leq 0.001$, for *CDKN2A* with r = 0.72 and $P \leq 0.001$, as well as for *PTEN* with r = 0.81 and $P \leq 0.001$

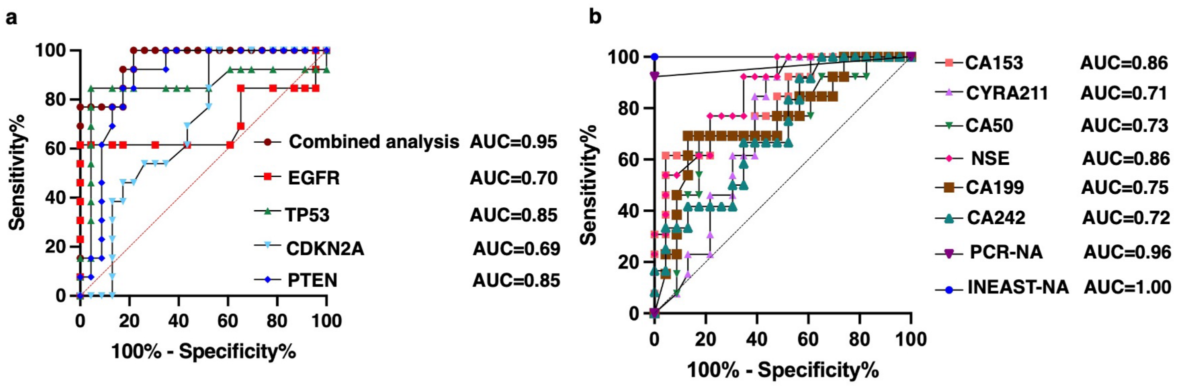

Supplementary Figure S8. ROC curves of PCR- and glycomarker-based bioassays. a. ROC curves demonstrated the PCR-based lung cancer diagnosis performance with the different cfDNA biomarkers; **b.** Glycosylated protein biomarkers and their combined diagnostic efficacy with cfDNA.

Currently, PCR was be recognized as the gold standard for nucleic acid detection and quantification including cfDNA. Therefore, PCR-based cfDNA bioassays were harnessed to quantify the collected clinical samples. Specifically, the PCR bioassays of *TP53* and *PTEN* demonstrated the best diagnostic efficacy as shown in **Figure S8, Supplementary Information**, achieving an AUC of 0.85. In contrast, *CDKN2A* and *EGFR* PCR bioassays exhibited diminished diagnostic efficacies with AUCs of 0.69 and 0.70 respectively. Notably, the combined analysis of four cfDNA with gold standard PCR bioassays resulted in an enhanced diagnostic performance, with an elevated AUC of 0.95. This results further demonstrated the primary benefit for increased accuracy in lung cancer diagnosis as shown in **Figure S8a, Supplementary Information**. The proposed INEAST bioassay demonstrated a competitive diagnostic performance with an AUC of 0.94 (**Figure 5f)** in comparison to the gold standard PCR approaches. It is noteworthy that the INEAST bioassay exhibited advantages over conventional gold standard PCR techniques regarding assay speed and ease-of-use.

Specifically as shown in **Figure S8b, Supplementary Information**, the AUCs of the combined bioassay, which considered both glycosylation and cfDNA biomarkers, reached 1.0 and 0.96, respectively, when using the INEAST- and PCR-based cfDNA bioassay results. In contrast, the AUC for solely PCR- and INEAST-based cfDNA analysis were found to be 0.95 and 0.94 respectively. Similar to individual cfDNA sequences, the assessment of any single glycomarkers has not resulted in an AUC exceeding 0.9. These findings further demonstrated that the accuracy of lung cancer diagnosis can be enhanced by the combined use of multiple cfDNA and glycosylation biomarkers.

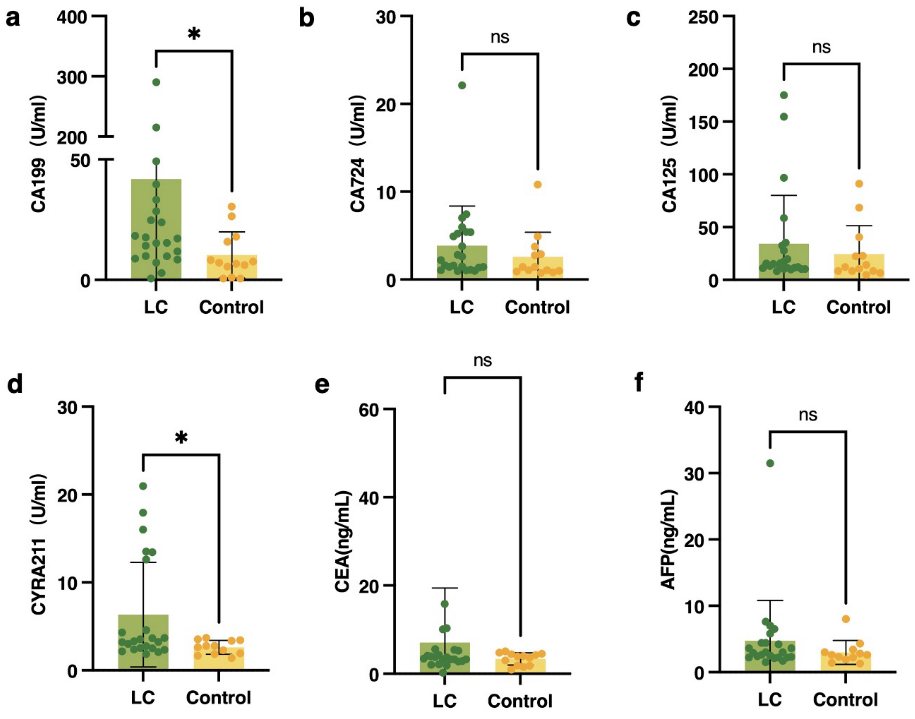

Supplementary Figure S9. Histograms for glycosylation-related biomarkers. The bar plots of expression levels for CA199 (**a**), CA724 (**b**), CA125 (**c**), CYRA211 (**d**), CEA (**e**) and AFP (**f**) for lung cancer patients and non-cancer patients. The error bars represent the SD measured from 3 different times.

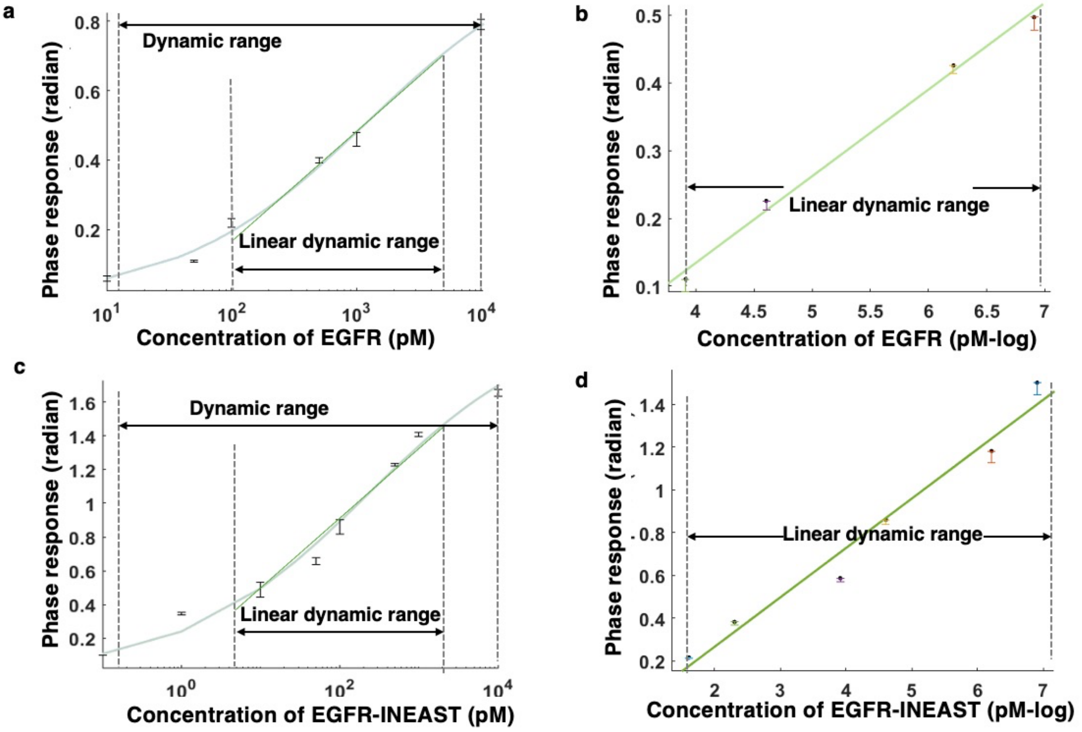

Supplementary Figure S10. The dynamic range of INEAST biosensing platform for enrichment and amplification *EGFR* cfDNA. a. The dynamic range for the enrichment of *EGFR* cfDNA: 12.87 pM ~ 10000 pM; **b.** The linear dynamic range for the enrichment of *EGFR* cfDNA: 75 pM ~ 500 pM; **c.** The dynamic range for the amplification of *EGFR* cfDNA: 0.04 pM ~ 10000 pM; **d.** The linear dynamic range for the amplification of *EGFR* cfDNA: 35 pM ~ 800 pM.

It is demonstrated that the dynamic range for the enrichment of *EGFR* cfDNA spans from 12.87 pM to 10,000 pM. The linear dynamic range for the enrichment of *EGFR* cfDNA was established between 750 pM and 5000 pM. The amplification process yielded a dynamic range for *EGFR* cfDNA amplification of 0.04 pM to 10,000 pM, while the linear dynamic range was established between 35 pM and 7,000 pM.

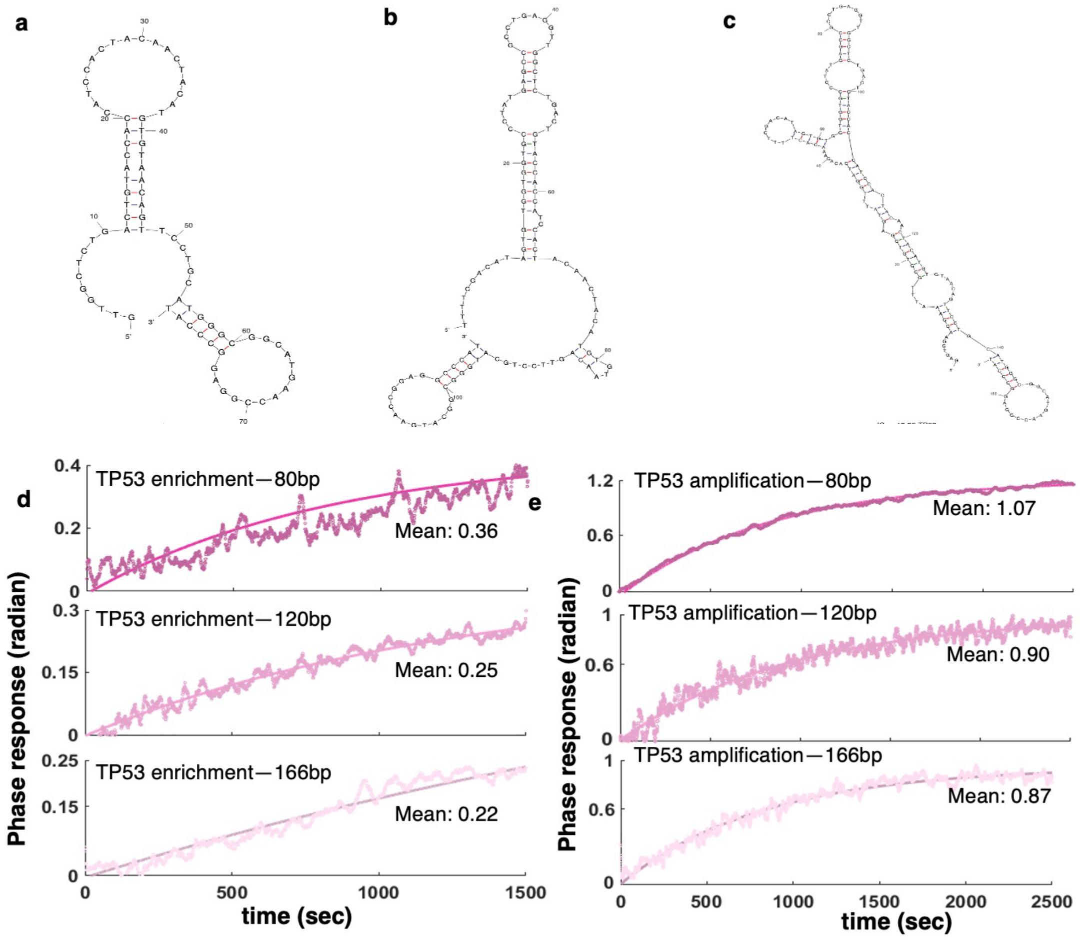

Supplementary Figure S11. Secondary structure prediction of the *TP53* cfDNA sequences via mfold (http://www.unafold.org/forum/) and the INEAST enrichment and amplification bioassay results toward three different *TP53*-cfDNA lengths. a. the secondary structure of cfDNA with a sequence length of 80 nt and the Gibbs energy was found to be ΔG = -8.18 kcal/mol; **b.** The secondary structure of cfDNA with an intermediate sequence length 120 nt and the Gibbs energy was found to be ΔG = -17.76 kcal/mol; **c.** The secondary structure of cfDNA with a full sequence length of 166 nt and the Gibbs energy was found to be ΔG = -19.95 kcal/mol; **d.** The real-time enrichment biosensing results with the INEAST bioassays for detecting the three different cfDNA length of 80 nt, 120 nt and 166 nt; **e.** The real time sensorgrams of the INEAST amplification bioassays for detecting the various cfDNA length of 80 nt, 120 nt and 166 nt.

The prolonged fragment size of cfDNA has negative effects on the hybridization efficiency by hinder the maintenance of a rigid structure of the nucleic acid strand. Generally, longer nucleic acid sequences tend to form more intricate secondary structures. This concept was further illustrated by simulating the secondary structures of the *TP53* cfDNA with various sequence lengths using the online DNA mfold tool (http://www.unafold.org/forum/). As illustrated in **supplementary Figure S11**, the long cfDNA sequences with 166 nt in length exhibited complex binding structure and high Gibbs energy of -19.95 kcal/mol, suggesting poor rigidity for biosensing application. To further investigated the INEAST biosensing performance toward different cfDNA fragment, we conducted comparison experiments on detecting three different *TP53* sequences with 80 nt, 120 nt, and 166 nt respectively as shown in **supplementary Figure S11**. It is noticed that the enrichment bioassay was slightly impacted by the elevated cfDNA length and the responses decreased by 38.9 % from 0.36 radian to 0.22 radian. In contrast, the amplification bioassay demonstrated better biosensing robustness toward different fragment lengths, as the amplification responses slightly decreased by 18.7 % from 1.07 radian to 0.87 radian. It is known that the DNA conformation, rigidity, and internal strand structure can be altered by manipulating temperature, electrostatics, π interactions, and hydrophobicity. Therefore, effective thermoplasmonic temperature control within the INEAST bioassay plays a vital role to minimize the impact of cfDNA length and hybridization efficiency.

Supplementary Table S1. Information of designed sequences for the INEAST bioassays.

Name	Sequence (5'-3')	Length (pb)	Melting temperature	Function
Circular-DNA (*CDKN2A*)	AGTCGGAAGTACTACTCTCTGTGTGTGAGAGTG GCGGGGTCGGCGCAGTTGATGCAGCTCCTCAGT AATAGTGTC	75	Tm: 89 °C	Padlock and Target sequence amplification template
cDNA-receptor (*CDKN2A*)	GTGTCCAGGAAGCCCTCCCGGTTTTTT	28	Tm: 69.1 °C	Capture
Template (*CDKN2A*)	CAACTGCGCCGACCCCGCCACTCTCACCCGACC CGTGCACGACGCTGCCCGGGAGGGCTTCCTGGA CACGCTG	73	Tm: 89 °C	Quantitative determination
Circular-DNA (*PTEN*)	AGTCGGAAGTACTACTCTCTGTGTGTAATAAATA TGCACATATCATTACACCAGTTCGTCCCTTTCCAT GCAGCTCCTCAGTAATAGTGTC	91	Tm: 87.1 °C	Target sequence amplification template

cDNA-receptor (*PTEN*)	GCCTCTTGTGCCTTTAAAAATTTGCTTTTTTT	25	Tm: 62.5 °C	Target sequence cyclization helper
Template (*PTEN*)	GGAAAGGGACGAACTGGTGTAATGATATGTGCA TATTTATTACATCGGGGCAAATTTTTAAAGGCAC AAGAGGCCCTA	78	Tm: 85.2 °C	Capture
Circular-DNA (*TP53*)	AGTCGGAAGTACTACTCTCTGTGTTAGTTGTAGT GGATGGTGGTACAGTCAGAGCCAATGCAGCTCC TCAGTAATAGTGTC	81	Tm: 88 °C	Quantitative determination
cDNA-receptor (*TP53*)	GGGCCTCCGGTTCATGCCGCTTTTTTT	20	Tm: 68.7 °C	Target sequence cyclization helper
Template (*PT53*)	TGGCTCTGACTGTACCACCATCCACTACAACTAC ATGTGTAACAGTTCCTGCATGGGCGGCATGAACC GGAGGCCCAT	78	Tm: 90.5 °C	Capture
Circular-DNA (*EGFR*)	AGTCGGAAGTACTACTCTCTGTGTGGCCAGCCC AAAATCTGTGATCTTGACATGCTGCATGCAGCTC	81	Tm: 89 °C	Quantitative determination

	CTCAGTAATAGTGTC-P			
cDNA-receptor (*EGFR*)	CTCCTTCTGCATGGTATTCTTTCTCTTC-TTTTTT	27	Tm: 69.1 °C	Capture
Template (*EGFR*)	GCAGCATGTCAAGATCACAGATTTTGGGCTGGC CAAACTGCTGGGTGCGGAAGAGAAAGAATACCA TGCAGAAGGAGGCA	81	Tm: 89 °C	Quantitative determination
PLP	TACTTCCGACTGACACTATTACT	23	Tm: 59.2 °C	Target sequence cyclization helper

Supplementary Table S2. The real-time enhancement of the localized thermoplasmonic heating effect for the INEAST biosensing process.

**Testing conditions**	**Detection time (sec)**	**Biosensing responses for concentration (radian)**		
		10 nM	1 nM	100 pM
**With PPT**	500	1.10	0.87	0.36
	1000	1.45	1.19	0.59
	1500	1.67	1.31	0.73
	2000	1.69	1.35	0.80
	2500	1.73	1.36	0.85
**Without PPT**				
	500	0.19	0.13	0.03
	1000	0.27	0.17	0.05
	1500	0.30	0.19	0.07
	2000	0.31	0.19	0.09
	2500	0.31	0.20	0.09

Supplementary Table S3. The information of PCR primers for quantitative *EGFR*, *TP53*, *CDKN2A* and *PTEN* analysis.

Gene	Primer sequence	Genomic coordinates of amplified region
EGFR	Forward: GCAGCATGTCAAGATCACAGAT	chr7: 55259486-55259565
	Reverse: TGCCTCCTTCTGCATGGTAT	
TP53	Forward: TGGCTCTGACTGTACCACCA	chr17: 7577529-7577606
	Reverse: ATGGGCCTCCGGTTCAT	
CDKN2A	Forward: ACCCTGGCTCTGACCATTC	chr9: 21971169-21971241
	Reverse: CAGCAGCTCCGCCACTC	
PTEN	Forward: CATGTTGCAGCAATTCACTGT	chr10: 89692868-89692942
	Reverse: CCGATGTAATAAATATGCACATATCATTAC	

Supplementary Table S4. The Hill equation-based regression analysis and the quantitative biosensing performance with the INEAST system.

	Biomarkers	Fitting formula	LoD	LoQ
**cfDNA hybridization/enrichment detection**	*EGFR*	$f(x)=0.95*(x/761.2)^{0.64}/1+ *(x/761.2)^{0.64}$	7748	27608
	TP53	$f(x)=0.594*(x/252.8)^{0.69}/1+ *(x/252.8)^{0.69}$	319	8380
	CDKN2A	$f(x)=0.8793*(x/319.3)^{0.63}/1+ *(x/319.3)^{0.63}$	1041	3058
	PTEN	$f(x)=0.913*(x/293.2)^{0.57}/1+ *(x/293.2)^{0.57}$	3022	8380
**cfDNA amplification-based detection**	*EGFR*	$f(x)=2.188*(x/301.6)^{0.35}/1+ *(x/301.6)^{0.35}$	24	175
	TP53	$f(x)=2.348*(x/504.3)^{0.43}/1+ *(x/504.3)^{0.43}$	24	445
	CDKN2A	$f(x)=3.216*(x/4942)^{0.29}/(1+x/4942)^{0.29}$	6	90
	PTEN	$f(x)=3.457*(x/5847)^{0.34}/1+ *(x/5847)^{0.34}$	12	30

Supplementary Table S5. cfDNA test methods and LoD.

Gene name	LoD	Method	Reference
AFP	20 ng/uL	cSMART	[2]
H37Rv	16 copies per gene	WATSON	[3]
HER2	3.5 copies	ddPCR	[4]
fDNA	10 fg/mL	NMDs beads-PCR	[5]
EGFR	8.48 ng/mL	TaqMan based TERT	[6]
single molecule cfDNA	1 copy/test	FL-enhancing PCR	[7]
**Our work**			
EGFR	24 copies number	INEAST	Our work
TP53	24 copies number	INEAST	Our work
CDKN2A	6 copies number	INEAST	Our work
PTEN	12 copies number	INEAST	Our work

Abbreviation: cSMART, Circulating Single-Molecule Amplification and Resequencing Technology; WATSON, Whole-genome Assay using Tiled Surveillance of Nucleic acids; ddPCR, droplet digital PCR; TERT, telomerase reverse transcriptase; FL-enhancing PCR, fluorescence-enhancing PCR.

Supplementary Table S6. The cut-off value of cfDNA in different tumor diagnosis.

Cancer type	cfDNA cut-off value	Reference
Glioma	349.22 ng/mL	[8]
Breast cancer	328.22 ng/mL	[8]
Hepatocellular carcinoma	30.00 ng/mL	[9]
Lung cancer	20.3 ng/mL	[10]
Oral cancer	368.27 ng/mL	[8]
Non-small cell lung cancer	41.46 ng/mL	[11]

Supplementary Table S7. Compared with the rolling circle amplification related methods.

Methods	Label	Detection time	LoD	Reference
RCA	methylene blue and acridine orange	2 h	1 copy/μL	[12]
RCA-CRISPR/Cas12a	G-quadruplex/hemin	45 min	0.3 fg/uL	[13]
RCA-SDR	FRET	2 h	0.03 nM	[14]
SPR-RCA	Cy5	250 min	13 pM	[15]
RCA	SYBR Green I	3 h	50 nM	[16]
MP-RCA	SYBR gold	3 h	qualitative experiment	[17]
PH-RCA	SYBR Green I	<30 min		[18]
C2CA-RCA	Magnetic nanoparticle	100 min	0.4 fM	[19]
INESAT	Label free	45 min	~0.04 pM	Our work

* RCA: rolling-circle-amplification; SDR: strand-displacement-reaction; SPR: surface plasmon resonance; PH-RCA: palindromic hyperbranched rolling circle amplification; C2CA: Circle-to-circle amplification.

Supplementary Table S8. The biosensing recoveries of the INEAST system.

TP53	**Detection value**	**Recovery**
1 nM	1.38 (1087 pM)	108.7%
100 pM	0.78 (99 pM)	99%
10 pM	0.33 (10.3 pM)	103%

Supplementary Table S9. Baseline information for the LC patient group and the control group.

	**Lung cancer group**	**Control group**	**P value**
Age	60.91±11.58	66.62±6.95	0.13
Gender (F*)	60.00%	53.8%	0.69
BMI	24.27±3.55	22.74±2.95	0.20
Smoking	56.60%	53.80%	0.88
Diabetes	30.43%	30.77%	0.81
Hypertension	56.52%	46.15%	0.56

*F refers to the percentage of female by gender

Briefly, a clear definition of the inclusion criteria for lung cancer patients and control subjects was provided in the **Methods** section. Specifically, the control group from high risk individuals presenting benign lung diseases, pulmonary nodules, or pulmonary shadow were selected in this work. Furthermore, we excluded patients who had previously undergone lung cancer surgery, chemotherapy, or those with complicating conditions such as diseases affecting the immune system. Additionally, the clinical baseline information has been summarized for both lung cancer patients and the control individuals involved in this study as demonstrated in **Table S9,**

Supplementary Information. The analysis focused on baseline characteristics including age, sex, smoking history, and chronic diseases, which indicated that the involved subjects demonstrated no significant differences in age, smoking history, or chronic diseases in this study.

Supplementary Table S10. The characterization of patients' cfDNA sequences in blood samples by sequencing method.

**Patient ID**	**cfDNA Gene**	**Health conditions of patient**	**Sequence (3'-5')**
4	*PTEN*	LC	TGTTCTGCGTGTCCAGGAAGCCCTCCCGGCAGCGTCTCACGTACG…GGAGTATAATAT GCACATATCATTACACCAGTTCGTCCCTTTCTAAG
5	*PTEN*	LC	TCTGCGTGTCCAGGAAGCCCTCCCGGCAGCGTCTCACGGAACG…GGGGCGAGTATAA TATGCACATATCATTACACCAGTTCGTCCCTTTCC
38	*PTEN*	Non-LC	TGGAATAGGGACGAACTGGTGTAATGATATGTGCATATTATACTCGTAT…GGGTGCATTT ATTAATCGGGGCAATTTTTAAGGCACAAGAGGCCCTAA
39	*PTEN*	Non-LC	CTAGAAAGGGACGAACTGGTGTAATGATATGTGCATATTATACTCGG…CGAAGCATTTA TTAATCGGGGCAATTTTTAAGGCACAAGAGGCCCTCAG
48	*PTEN*	Non-LC	CTAGAAAGGGACGAACTGGTGTAATGATATGTGCATATTATACTCGG…CGAAGCATTTA TTAATCGGGGCAAATTTTTAAAGGCACAAGAGGCCCTCAG

71	*PTEN*	LC	GGGTGCATTTATTACATCGGGGCAATTTTTAAGGCACAAGAGGCCCTAA…GGGTGCATTTATTACATCGGGGCAATTTTTAAGGCACAAGAGGCCCTAA
4	*EGFR*	LC	CTATAGGGCCTCTTGTGCCTTAAAAATTGCCCCGATGTAATAAAAGCGATGC…TGACTCACTGCTGGGTGCGGAGAGAAGATACCATGCAGAAGGAGGCA
5	*EGFR*	LC	TAGGGCCTCTTGTGCCTTAAAAATTGCCCCGAGTAATAAATGCACGC…TTGACAGCAGTTTGGCCAGCCCAAATCTGTGATCTTGACATGCTAGCAGA
38	*EGFR*	Non-LC	GCAGCATGTCAAGATCACAGATTTGGGCTGGCCAAACTGCTGGTCGAG…CTCGACCAGCAGTTTGGCCAGCCCAAATCTGTGATCTTGACATGCTGC
39	*EGFR*	Non-LC	GCAGCATGTCAAGATCACAGATTTGGGCTGGCCAAAGCG…TGGGTGCGGAGAGAAGATACCATGCAGAAGGAGGCA
48	*EGFR*	Non-LC	TCTTGCTAGCATGTCAAGATCACAGATTTGGGCTGGCCAAACTGCTGTCACT…GGGCTTACTGCTGGGTGCGGAGAGAAGATACCATGCAGAAGGAGGCA
71	*EGFR*	LC	CTTGCAGCATGTCAAGATCACAGATTTGGGCTGGCCAAACTGCTGGTCA…GGCTTACTGCTGGGTGCGGAAGAAGAAGATACCATGCAGAAGGAGGCA

4	*CDKN2A*	LC	TCTAATGGGCCTCCGGTTCATCCGCCCATGCAGGAACTGTACACAGAG…TGGCTGTGCGGGTCGGGTGAGAGGGCGGGGTCGGCGCAGTAGA
5	*CDKN2A*	LC	CTCGAATGGGCCTCCGGTTCATGCCGCCCATGCAGGAACTGTATAGTAGAA…TCCATCGTCCGGGTCGGGTGAGTATGGCGGGGTCGGCGCAGTAGA
38	*CDKN2A*	Non-LC	TCTACTGCGCCGACCCCGCCATCTCACCCGACCCTCACAATCCTC…CGAGAGACGCTGCCGGGTAAGGGCTTCCAGGACACGCACGA
39	*CDKN2A*	Non-LC	TCTACTGCGCCGACCCCGCCATTCTCACCCGACCCGCCGATCCA…AGTACGGTAGACGCTGCCGGGGAGGGCTTCCTGGACACGCTGA
48	*CDKN2A*	Non-LC	TCTACTGCGCCGACCCCGCCCTATCTCACCCGACCCGGATGCCCAC…ACGGTAGTACGCTGCCGGGTAGGGCTTCCTGGACACGCTACGA
71	*CDKN2A*	LC	TCAACTGCGCCGACCCCGCCATCTCACCCGACCCGCCGGCCAC…ACGTACGATCGACGCTGCCCGGGAGGGCTTCCTGGACACGCTAAAA
9	*CDKN2A*	LC	TCAACTGCGCCGACCCCGCCATTTCACCCGACCCGCCATGAGCGGGGTC…TTCGTCCGTGAGACGCTGCCGGGAGGGCTTCCTGGACACGCT

17	*CDKN2A*	LC	CTCTTACTTACGCCGAACCCCCGAAATCTCACCCGACCCGTGCACAACG…ATAAATAT ATCGTCCGCGAGACGCTGCCGGGTAGGGCTTCCTGGACACGCAGAGA
13	*CDKN2A*	LC	TAATGTATTCGACACCCCGAAACGTCACCCGACCTTCACAACTGGCGTG…ATATATTTG TTCGAAGCGCCGACGCTGCCGGGTAAGGGCTTCCTGGACACTGCACA
27	*CDKN2A*	Non-LC	TATATCCGACTGCGCCGACCCCCGAAACCTCACCCAACCCGGCAGGAGTCGG…GCGT ACGTGCCGAAGCTGCCGGGTGGGGCTTCCAGGACACGCTCGAG
25	*CDKN2A*	Non-LC	TGGATTCCCACTTCCGAAACGTCCCTCGACCTGGCTGAATCCGAG…TCGCATAGATGA ATACGCTTCCCGGGTGGGTACCTGGACACGCAGGA
14	*CDKN2A*	LC	TTCTCCACACTACGCCGACCCCCCAAATCTCACCCGACCCGGCACACCCCC…TCGTTC CATGCACTAAGCTGTCCGGGTGGGCTTCCAGGACACGCACGAGCA
28	*CDKN2A*	Non-LC	TCGGACTTGCGCCGACCCCGCCAATTCACCCGACCCTCACAGC…TCGAAAGATGAATA CGCTTCCCGGGTCGGTACCTAGAACGCTGA
4	*TP53*	LC	TGCCTCCTTCTGCATGGTATCTTCTCTCCGCACCCAGCAGTGAGCAC…GGTGATGTTAC ATGTAGTTGTAGTGGTGGTGGTACAGTCAGAGCCA

5	*TP53*	LC	TGCCTCCTTCTGCATGGTATCTTCTCTCCGCACCCAGCAGTGAGCAC…ATGGAGCGCT TCCATGCTAGTTGTAGTGGATGGTGGTACAGTCAGAGCCAA
38	*TP53*	Non-LC	TTGGCTCTGACTGTACCACCACCACTACAACTACATGTAACATCGTC…AAGTCTCTGCT ACAGTTCTGCATGGGCGGCATGAACCGGAGGCCCATCGAG
39	*TP53*	Non-LC	TTGGCTACTGACTGTACCACCATCCACTACAACTAGCATGGAAACC…AAACTGCTGCT ACAGTTCCTGCATGGGCGGCATGAACCGGAGGCCCATT
48	*TP53*	Non-LC	TGGCTCTGACTGTACCACCATCCACTACAACTACATGTAGCGACTCC…AAACTCTGCT ACAGTTCCTGCATGGGCGGCATGAACCGGAGGCTACATACGAG
71	*TP53*	LC	TGGCTCTGACTGTACCACCATCCACTACAACTAGCATGTAAGCGCTC…AAACTGTATAC AGCTTCCTGCATGGGCGGCATGAACCGGAGGCCCATCGA
9	*TP53*	LC	TAGCGTCTGACTGTACCTCGAACCACGACAACTACATTTACAGAGCAGTCAAGCTGGC …TCTACTGTGTACAGTTCCTGCATGGGCGGGATGAACCGGAGGCCCTTGAG
17	*TP53*	LC	TTGGCTCTGACTGTACCACCACCACTACAACTACATGTAACATCGCCG…AACTCTGTG TACAGTTCCTGCATGGGCGGATGAACCGGAGGCCCATAAGAG
13	*TP53*	LC	TTGCGCTGACTGTCCACAATCCACTACAACTACAGTAACATTACGCG…ACTCTGTGTA

			CAGTTCCTGCATGGGCGGATGAACCGGAGGCCCATAAG
27	*TP53*	Non-LC	AGCGCTGACTGTCCACAACCACTACAACTACAGTAACAGTACGCCG…ATTCTGTGTAA GTTCCTGGCTGGGCGGCTGAACCGGCGGCTAAACAAG
25	*TP53*	Non-LC	CAAGCTTCTGACTGTCCTCAACCCACTACAACTACATGTAACATCGC…CCTTTATGTGT AAGTTCCTGCATGGGCGGATGAACCGGAGGCCCTA
14	*TP53*	LC	TTGTCTCTGACTGTACCACCACCACTACAACTACATGTAACATCACAC…CACTCTGTGT ACAGTTCCTGCATGGGCGGATGAACCGGAGGCCCATTAG

Supplementary Table S11. Evaluation of the INEAST diagnostic performance with receiver operating characteristic curves.

**Biomarkers**	**Specificity**	**Sensitivity**	**AUC**	**Cut-off**
EGFR	78.62	92.31	0.86	0.71
TP53	91.30	84.62	0.86	0.76
CDKN2A	47.83	76.92	0.58	0.25
PTEN	100	76.92	0.82	0.77
**Combined diagnosis**	73.91	100	0.94	0.74

Supplementary Table S12. The bioassay results of the selected glycosylated proteins and their corresponding functions for cancer diagnosis.

Glycosylated protein	Lung cancer	Control group	P value	Sensitivity (%)	Specificity (%)	Function*
**CA242 (U/mL)**	8.03	3.68	0.04	37.50	89.47	Pancreatic cancer/Lung cancer [20]
**CA50 (U/mL)**	10.73	4.88	<0.01	75.00	89.47	Gastro-intestinal cancer/ Pancreatic cancer [21]
**CA19-9 (U/mL)**	41.82	10.32	0.04	69.23	86.96	Colorectal cancer/Lung cancer Gallbladder cancer [20b, 22]
**CA724 (U/mL)**	3.86	2.58	0.36	30.77	91.30	Pancreatic cancer[23]
**CA125 (U/mL)**	34.20	24.53	0.49	30.77	95.65	Pancreatic cancer[24]
**CYFRA211 (ng/mL)**	6.33	3.14	0.04	33.33	95.65	Lung cancer[25]
**NSE (ng/mL)**	17.63	8.68	0.04	53.85	95.65	Lung cancer[26]
**CEA (ng/mL)**	7.05	3.35	0.29	46.15	52.17	Lung cancer/ Colorectal cancer [25, 27]

* The references provide detailed information about the glycomarkers in specific tumors.

These glycosylation biomarkers have been currently recognized as standard tumor screening biomarkers in clinical practice. Glycosylated proteins are crucial in the diagnosis of tumors, particularly for early detection. One notable example is Neuron-Specific Enolase (NSE), which serves as a biomarker for the diagnosis, staging, and

monitoring of small cell lung cancer (SCLC) [28]. CEA is an oncofetal glycoprotein and commonly served as biomarker for routine tumor screening [29]. It plays a role in cellular adhesion and is notably expressed in endodermal epithelial tumors. According to **Supplementary Table S12**, the sensitivity and specificity of CEA were found to be 46.15% and 56.17%, respectively. CA19-9 typically expressed in normal human epithelial tissues such as those found in the pancreatic duct, bile duct, gall bladder, stomach, salivary gland, bronchus, prostate, colon, rectum, and uterus [30]. In our lung cancer cohort, CA19-9 exhibited a sensitivity of 69.23% and specificity of 86.96%. This suggests that CA19-9 may be more effective than both NSE and CEA in terms of sensitivity for detecting lung cancer. Research indicates that when NSE is used alongside Carcinoembryonic Antigen (CEA), the specificity of CA15-3 as a biomarker for breast cancer can be enhanced to 95% [31]. In addition, reliance on single markers or insufficiently characterized glycosylated proteins demonstrated limitations on tumor differentiation. This has prompted researchers to explore combinations of various glycosylated protein markers to enhance diagnostic precision. Wen et al. reported that the expression levels of ten serum tumor markers, including CYFR21, CEA, NSE, SCC, CA15-3, CA19-9, CA125, CA50, CA242, and CA724 [25]. Specifically, the AUC was 0.854 for a panel consisting of four biomarkers, i.e., CYFR21, CEA, NSE, and SCC. By further considering CA125 and CA15-3, the AUC improved to 0.875. The most comprehensive panel containing all ten markers achieved an AUC of 0.884. These findings inspired us that the combinational approaches with multiple glycomarkers enhanced the specificity and sensitivity of tumor diagnosis. Therefore, multiple glycomakers and cfDNA factors were considered in our studies.

Supplementary Table S13. The dynamic ranges of the INEAST-based cfDNA bioassays.

Gene	INEAST	Dynamic range (pM)	Linear dynamic range (pM)
EGFR		12.87 ~ 10000	750 ~ 5000
TP53	**Enrichment**	0.53 ~ 10000	550 ~ 5000
CDKN2A		1.73 ~ 10000	650 ~ 5000
PTEN		5.02 ~ 10000	350 ~ 6000
EGFR		0.04 ~ 10000	35 ~ 7000
TP53	**Amplification**	0.04 ~ 10000	25 ~ 7800
CDKN2A		0.01 ~ 10000	15 ~ 8000
PTEN		0.02 ~ 10000	30 ~ 8500

Reference

[1] a) G. G. Qiu, Z. B. Gai, Y. L. Tao, J. Schmitt, G. A. Kullak-Ublick, J. Wang, *Acs Nano* **2020**, *14* (5), 5268, https://doi.org/10.1021/acsnano.0c02439; b) G. Qiu, Z. Gai, L. Saleh, J. Tang, T. Gui, G. A. Kullak-Ublick, J. Wang, *Acs Nano* **2021**, *15* (4), 7536, https://doi.org/10.1021/acsnano.1c00957.

[2] T. Wu, R. Fan, J. Bai, Z. Yang, Y. S. Qian, L. T. Du, C. Y. Wang, Y. C. Wang, G. Q. Jiang, D. Zheng, X. T. Fan, B. Zheng, J. F. Liu, G. H. Deng, F. Shen, H. P. Hu, Y. N. Ye, Q. Z. Zhang, J. Zhang, Y. H. Gao, J. Xia, H. D. Yan, M. F. Liang, Y. L. Yu, F. M. Sun, Y. J. Gao, J. Sun, C. X. Zhong, Y. Wang, H. Wang, F. Kong, J. M. Chen, H. Wen, B. M. Wu, C. X. Wang, L. Wu, J. L. Hou, X. L. Liu, H. Y. Wang, L. Chen, *J Hematol Oncol* **2023**, *16* (1), 1, https://doi.org/10.1186/s13045-022-01396-z.

[3] S. G. Thakku, J. Lirette, K. Murugesan, J. Chen, G. Theron, N. Banaei, P. C. Blainey, J. Gomez, S. Y. Wong, D. T. Hung, *Nat Commun* **2023**, *14* (1), 1803, https://doi.org/10.1038/s41467-023-37183-8.

[4] E. Boldrin, M. Mazza, M. A. Piano, R. Alfieri, I. M. Montagner, G. Magni, M. C. Scaini, L. Vassallo, A. Rosato, P. Pilati, A. Scapinello, M. Curtarello, *Cancers (Basel)* **2022**, *14* (9), https://doi.org/10.3390/cancers14092180.

[5] I. Vogt-Moykopf, T. Fritz, G. Meyer, H. Bulzerbruck, G. Daskos, *Int Surg* **1986**, *71* (4), 211.

[6] A. M. Mazurek, T. Rutkowski, A. Fiszer-Kierzkowska, E. Malusecka, K. Skladowski, *Oral Oncol* **2016**, *54*, 36, https://doi.org/10.1016/j.oraloncology.2015.12.002.

[7] M. Iwanaga, T. Hironaka, N. Ikeda, T. Sugasawa, K. Takekoshi, *Nano Lett* **2023**, *23* (12), 5755, https://doi.org/10.1021/acs.nanolett.3c01527.

[8] S. Kumari, S. Mishra, N. Husain, T. Verma, V. Tiwari, M. Kaif, A. Agarwal, M. Rastogi, S. Shukla, A. A. Sonkar, *Indian J Pathol Microbiol* **2022**, *65* (1), 93, https://doi.org/10.4103/IJPM.IJPM_474_20.

[9] L. Yan, Y. Chen, J. Zhou, H. Zhao, H. Zhang, G. Wang, *Int J Infect Dis* **2018**, *67*, 92, https://doi.org/10.1016/j.ijid.2017.12.002.

[10] J. M. Gonzalez de Aledo-Castillo, A. Arcocha, I. Victoria, A. I. Martinez-Puchol, C. Sanchez, P. Jares, G. F. Rodriguez, N. Vinolas, R. Reyes, N. Reguart, J. A. Puig-Butille, *J Thorac Dis* **2021**, *13* (3), 1658, https://doi.org/10.21037/jtd-20-3142.

[11] W. W. Peng, Y. Liu, H. H. Sha, S. D. Wen, Y. Fang, G. R. Zhou, *BMC Pulm Med* **2023**, *23* (1), 348, https://doi.org/10.1186/s12890-023-02586-2.

[12] T. Chaibun, J. Puenpa, T. Ngamdee, N. Boonapatcharoen, P. Athamanolap, A. P. O'Mullane, S. Vongpunsawad, Y. Poovorawan, S. Y. Lee, B. Lertanantawong, *Nat Commun* **2021**, *12* (1), 802, https://doi.org/10.1038/s41467-021-21121-7.

[13] Y. L. Liu, L. R. Ma, W. J. Liu, L. Y. Z. Xie, Q. Wu, Y. W. Wang, Y. Zhou, Y. H. Zhang, B. N. Jiao, Y. He, *J Agr Food Chem* **2023**, *71* (11), 4736, https://doi.org/10.1021/acs.jafc.2c07965.

[14] X. Peng, W. B. Liang, Z. B. Wen, C. Y. Xiong, Y. N. Zheng, Y. Q. Chai, R. Yuan, *Anal Chem* **2018**, *90* (12), 7474, https://doi.org/10.1021/acs.analchem.8b01015.

[15] K. Schmidt, S. Hageneder, B. Lechner, B. Zbiral, S. Fossati, Y. Ahmadi, M. Minunni, J. L. Toca-Herrera, E. Reimhult, I. Barisic, J. Dostalek, *Acs Appl Mater Inter* **2022**, *14* (49), 55017, https://doi.org/10.1021/acsami.2c14500.

[16] Y. Ye, Y. Lin, Z. L. Chi, J. S. Zhang, F. Cai, Y. Z. Zhu, D. P. Tang, Q. Q. Lin, *Peerj* **2022**, *10*, https://doi.org/ARTN e14257
10.7717/peerj.14257.

[17] R. Kumari, J. W. Lim, M. R. Sullivan, R. Malampy, C. Baush, I. Smolina, H. Robin, V. V. Demidov, G. S. Ugolini, J. R. Auclair, T. Konry, *Diagnostics* **2022**, *12* (9), https://doi.org/ARTN 2252
10.3390/diagnostics12092252.

[18] J. Song, Y. Ju, S. Kim, H. Kim, H. G. Park, *Chem Commun* **2022**, *58* (45), 6518, https://doi.org/10.1039/d2cc01370c.

[19] B. Tian, F. Gao, J. Fock, M. Dufva, M. F. Hansen, *Biosens Bioelectron* **2020**, *165*, https://doi.org/ARTN 112356
10.1016/j.bios.2020.112356.

[20] a) J. E. Macgregor, S. M. Moss, D. M. Parkin, N. E. Day, *Br Med J (Clin Res Ed)* **1985**, *290* (6481), 1543, https://doi.org/10.1136/bmj.290.6481.1543; b) Y. F. Wang,

F. L. Feng, X. H. Zhao, Z. X. Ye, H. P. Zeng, Z. Li, X. Q. Jiang, Z. H. Peng, *World J Gastroenterol* **2014**, *20* (14), 4085, https://doi.org/10.3748/wjg.v20.i14.4085; c) H. Dou, G. Sun, L. Zhang, *Prog Mol Biol Transl Sci* **2019**, *162*, 229, https://doi.org/10.1016/bs.pmbts.2018.12.007.

[21] O. Nilsson, C. Johansson, B. Glimelius, B. Persson, B. Norgaard-Pedersen, A. Andren-Sandberg, L. Lindholm, *Br J Cancer* **1992**, *65* (2), 215, https://doi.org/10.1038/bjc.1992.44.

[22] a) G. Liu, J. Liu, W. Ren, L. Yang, Q. Guo, H. Ye, *Medicine (Baltimore)* **2023**, *102* (47), e35949, https://doi.org/10.1097/MD.0000000000035949; b) K. Bjorkman, H. Mustonen, T. Kaprio, H. Kekki, K. Pettersson, C. Haglund, C. Bockelman, *Tumour Biol* **2021**, *43* (1), 57, https://doi.org/10.3233/TUB-200069.

[23] H. Liang, X. Yang, *J Oncol* **2022**, *2022*, 7742760, https://doi.org/10.1155/2022/7742760.

[24] Y. Chen, S. G. Gao, J. M. Chen, G. P. Wang, Z. F. Wang, B. Zhou, C. H. Jin, Y. T. Yang, X. S. Feng, *Cell Biochem Biophys* **2015**, *71* (3), 1287, https://doi.org/10.1007/s12013-014-0345-2.

[25] Z. Wen, Y. Huang, Z. Ling, J. Chen, X. Wei, R. Su, Z. Tang, Z. Wen, Y. Deng, Z. Hu, *Dis Markers* **2020**, *2020*, 4716793, https://doi.org/10.1155/2020/4716793.

[26] L. Yao, Y. Li, Q. Wang, T. Chen, J. Li, Y. Wang, L. Zhang, L. Su, L. Li, Q. Lou, F. Li, J. Zhao, J. Gao, J. Gao, H. Li, *J Cancer* **2023**, *14* (10), 1904, https://doi.org/10.7150/jca.85846.

[27] a) J. B. Pan, Y. H. Hou, G. J. Zhang, *Asian Pac J Cancer Prev* **2013**, *14* (2), 695, https://doi.org/10.7314/apjcp.2013.14.2.695; b) H. Rao, H. Wu, Q. Huang, Z. Yu, Z. Zhong, *Clin Lab* **2021**, *67* (4), https://doi.org/10.7754/Clin.Lab.2020.200828.

[28] A. S. Babkina, M. A. Lyubomudrov, M. A. Golubev, M. V. Pisarev, A. M. Golubev, *Int J Mol Sci* **2024**, *25* (9), https://doi.org/10.3390/ijms25095040.

[29] C. Hall, L. Clarke, A. Pal, P. Buchwald, T. Eglinton, C. Wakeman, F. Frizelle, *Ann Coloproctol* **2019**, *35* (6), 294, https://doi.org/10.3393/ac.2019.11.13.

[30] M. Dietel, H. Arps, R. Klapdor, S. Muller-Hagen, M. Sieck, L. Hoffmann, *J Cancer Res Clin Oncol* **1986**, *111* (3), 257, https://doi.org/10.1007/BF00389242.

[31] M. Rubach, J. J. Szymendera, J. Kaminska, M. Kowalska, *Int J Biol Markers* **1997**, *12* (4), 168, https://doi.org/10.1177/172460089701200406.
